# The Ca_V_2α1 EF-hand F helix tyrosine, a highly conserved locus for GPCR inhibition of Ca_V_2 channels

**DOI:** 10.1038/s41598-018-21586-5

**Published:** 2018-02-19

**Authors:** Tyler W. Dunn, Xiaotang Fan, Ariel R. Ase, Philippe Séguéla, Wayne S. Sossin

**Affiliations:** 1 Department of Neurology and Neurosurgery, Montreal Neurological Institute, McGill University, Montreal, Quebec, H3A 2B4 Canada; 2 Department of Neurology and Neurosurgery, Montreal Neurological Institute, Alan Edwards Centre for Research on Pain, McGill University, Montreal, Quebec, H3A 2B4 Canada

## Abstract

The sensory neuron of *Aplysia californica* participates in several forms of presynaptic plasticity including homosynaptic depression, heterosynaptic depression, facilitation and the reversal of depression. The calcium channel triggering neurotransmitter release at most synapses is Ca_V_2, consisting of the pore forming α1 subunit (Ca_V_2α1), and auxiliary Ca_V_β, and Ca_V_α2δ subunits. To determine the role of the Ca_V_2 channel in presynaptic plasticity in *Aplysia*, we cloned Aplysia Ca_V_2α1, Ca_V_β, and Ca_V_α2δ and over-expressed the proteins in Aplysia sensory neurons (SN). We show expression of exogenous Ca_V_2α1 in the neurites of cultured *Aplysia* SN. One proposed mechanism for heterosynaptic depression in *Aplysia* is through inhibition of Ca_V_2. Here, we demonstrate that heterosynaptic depression of the Ca_V_2 calcium current is inhibited when a channel with a Y-F mutation at the conserved Src phosphorylation site is expressed, showing the strong conservation of this mechanism over evolution. We also show that the Y-F mutation reduces heterosynaptic inhibition of neurotransmitter release, highlighting the physiological importance of this mechanism for the regulation of synaptic efficacy. These results also demonstrate our ability to replace endogenous Ca_V_2 channels with recombinant channels allowing future examination of the structure function relationship of Ca_V_2 in the regulation of transmitter release in this system.

## Introduction

*Aplysia* pleural sensory neurons are body wall sensory neurons involved in the defensive withdrawal reflexes of the animal and the locus for a variety of forms of synaptic plasticity^1,2^. Serotonin released with a noxious stimulus increases sensory neuron excitability and transmitter release^3–5^. Conversely, dopamine, FMRFamide, and activation of 5HT1 receptors reduce sensory neuron excitability and transmitter release leading to heterosynaptic depression^6–11^. As is the case at most synapses, transmitter release at *Aplysia* sensory neurons is triggered by voltage-gated calcium entry through Ca_V_2 calcium channels^6^. Aplysia pleural sensory neurons in culture have both Ca_V_1 and Ca_V_2 high-voltage-activated (HVA) currents and do not appear to express a low-voltage-activated calcium current (Ca_V_3 or Na_V_2), allowing isolation of the Ca_V_2 current with the block of the Ca_V_1 current with nifedipine^11^. With the exception of the vertebrates, bilaterians have a single gene coding the pore forming Ca_V_2 alpha 1 subunit (Ca_V_2α1). As there is evidence that the *Aplysia* Ca_V_2 current is regulated by dopamine^10,11^, FMRFamide^6^, and bidirectionally with serotonin (depending on the identity of the 5HT receptors activated^11,12^) we cloned the *Aplysia* Ca_V_2α1 along with the accessory subunits Ca_V_β and Ca_V_α2δ to further investigate this modulation.

The inhibition of the Ca_V_2 calcium current with G-protein coupled receptor (GPCR) activation is well documented. The G-protein Gβγ subunit can inhibit the channel directly through a thoroughly studied mechanism termed voltage-dependent (VD)-inhibition as the inhibition can be relieved with strong membrane depolarization^13^. However, VD-inhibition does not appear to occur with invertebrate Ca_V_2 channels^11,14^. Rapid Ca_V_2 inhibition can also occur with GPCR activation through downstream Src kinase activity^15,16^. Src kinase phosphorylation of the Ca_V_2α1 C-terminal EF-hand Y1747 is involved in the voltage-independent (VI)-inhibition with μ-opioid receptor activation^15,17,18^ and with GABA_B_ receptor activation^15,19^. Here we show that the Y1747 residue is highly conserved, found in most Ca_V_2 sequences, and point mutation of the Y1747 ortholog to phenylalanine in the *Aplysia* Ca_V_2α1 (Y1501F) reduces the inhibition of the calcium transient with dopamine and 5HT1A activation measured with fluorescence imaging. Furthermore, Ca_V_2α1 Y1501F expression in presynaptic sensory neurons reduced the heterosynaptic depression at sensory to motor neuron synapses with 5HT1A activation. This indicates that the VI-inhibition of the Ca_V_2 current through Src kinase phosphorylation of the F-helix EF-hand of Ca_V_2α1 is a physiologically important and highly conserved mechanism of Ca_V_2 regulation.

Functional expression of the exogenous, RFP-tagged Ca_V_2α1 subunits in cultured sensory neurons required at least 48 h of expression, evidenced by the inability of the Y1501F mutant to affect the inhibition of 5HT1A activation with only 24 h expression. The block of the inhibition with Y1501F expression appears to be complete at 48 h, indicating near complete substitution of the endogenous alpha 1 subunits with recombinant subunits at this time point.

## Results

**Figure 1:**
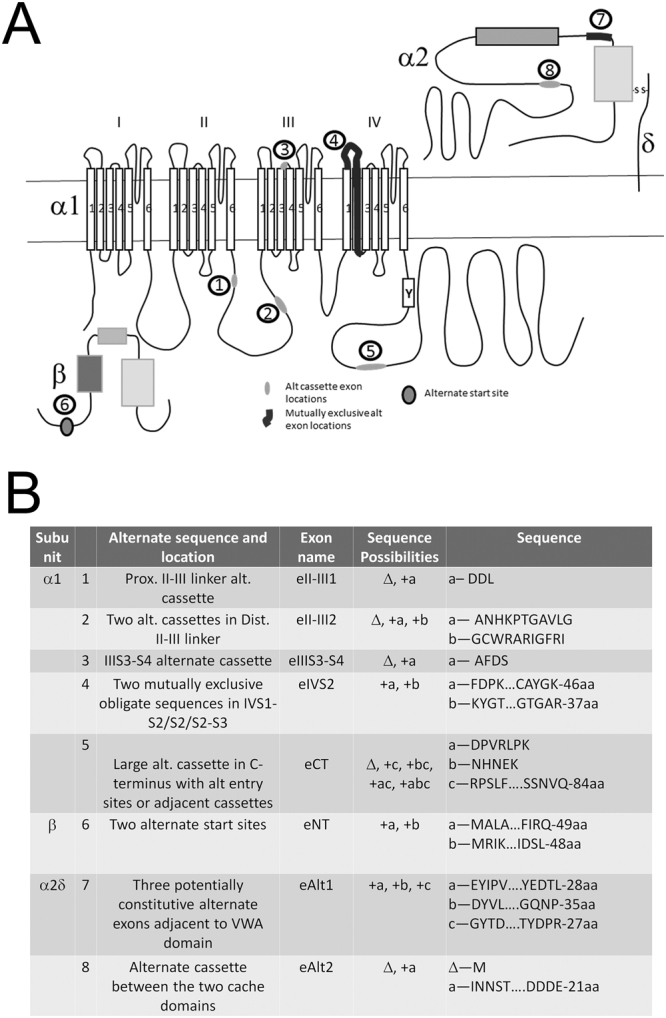
*Aplysia* Ca_V_2α1, Ca_V_β, and Ca_V_ α2δ alternate sequence locations from the available transcriptome data. (**A**) Cartoon layout of the *Aplysia* Ca_V_2α1 subunit showing the four (I–IV) domain repeats, each with 6 transmembrane spanning regions. The relative locations of four alternate sequence inserts indicative of alternate cassette exons are highlighted in light grey ovals and numerically labeled; two in the domain I-II linker loop (1&2), one in the C-terminus (5), and one in an extracellular IIIS3-S4 linker (3). The location of an alternate sequence in a constitutive region of domain IV is highlighted in dark, thick line (4). The location of the EF-hand with Y1501 is labeled with a Y. The cytosolic Ca_V_β subunit has at least two potential isoforms generated with an alternate start site producing subunits with distinct N-termini (6). The extracellular α2 subunit also has a variety of isoforms with two regions coding alternate sequences; the first with three potential sequences (7) and the second site is an alternate cassette exon (8). (**B**) Table of alternate sequence locations, isoform variations, and specific isoform sequences for the three cloned *Aplysia* Ca_V_ subunits.

We have cloned the pore forming subunit of the Ca_V_2 calcium channel from *Aplysia californica* (Ca_V_2 alpha 1 subunit- Ca_V_2α1) using primers designed from searching the *Aplysia* transcriptosome (www.aplysiagenetools.org) for sequences with homology to the cloned *Lymnaea* Ca_V_2 channel. Partial sequences were identified on distinct transcripts, however, several highly repetitive and CG rich regions probably prevented assembly of the entire message. Despite this, we were able to assemble a complete Ca_V_2α1 using PCR (Fig. 1-KY705237). Using the same strategy but with proteins from the *Lottia* genome we identified and cloned the Ca_V_β subunit (KY705239) and Ca_V_α2δ subunits (KY705238) sequences. Comparing the *Aplysia* Ca_V_2α1 sequence to the available genomic and transcriptome data for *Aplysia*, reveals at least five sites with alternate sequences (Fig. 1; Supplemental Information) Four of the five appear to be alternate cassette exons and one a pair of mutually exclusive obligate exons. There are two Ca_V_β transcriptional start sites generating two distinct 5′ ends (Fig. 1; Supplemental Information). We also found two alternatively spliced regions in the *Aplysia* Ca_V_α2δ Fig. 1; Supplemental information). Thus, while there are only single genes encoding the Ca_V_2 subunits, through alternative start sites and alternative splicing, there may be a wide variety of channels expressed in distinct neurons in *Aplysia* (Fig. 1B). Many of these splice sites are conserved in other Gastropods (oyster, *Crassostrea*), in other Molluscs (Octopus, Cephalopod), and in some cases in Chordates as well based on transcriptome data and previous publications (Table 1). The alternative start site for the beta subunit is conserved in most Bilaterians (Table 1).

**Table 1:** Conservation of the Aplysia calcium channel splice sites in the Gastropod *Crassostrea gigas*, the Mollusc *Octopus bimaculoides*, the Protostome *Drosophila m*., and vertebrates.

Splice	Location	Organisms With splice	Accession number	Sequence/Reference
CaV2 alpha 1 DDL	II-III Linker (proximal)	Crassostrea	XP_011419167.1 (no splice); all of the rest have the sequence	HLEEVRQDVHNQFTDHSQ
		Octopus	KOF72121.1 (no splice); all the rest have the sequence	EEEAAEKEKL
		vertebrates CaV2.2 & 2.3	Very near to e18a	^51^
ANHKPTGAVLG GCWRARIGFRI	II-III linker (Distal)	Crassostrea	XP_011419165.1 Most have no splice, above has insert:	ENNDLVNNIPR
AFDS	III S3–4	Crassostrea	XP_011419175.1	NTNVPS
		vertebrates CaV2.1 & 2.2	e24a	^52^
FDP….CAYGK KYGT…GTGAR	IVS2	Crassostrea	XP_011419166.1	FEGMS…LIGCGK
		Crassostrea	XP_011419175.1	FSNKS..LAFGIK
		Octopus	KOF72118.1	FYVNP…LGFGLK
		Octopus	XP_014784009.1	YNGSS…MGFGIR
Splice before Large insert DPVRLPK+/−NHNEK	C-terminus	Crassostrea	XP_011419171.1	VEDKEE
		Octopus	KOF72120.1	Large insert (LFWED…NQRP)
		Drosophila	NP_001259477.1	RKKL…VEEP
		Drosophila	NP_001245636.1	KQSF…RSVR
Alt start for beta subunit		Crassostrea	XP_011429594.1	
		Crassostrea	XP_011429598.1	
		Octopus	XP_014776325.1	
		Octopus	XP_014776323.1	
		vertebrates		^53^
		Drosophila	NP_995685.3	
		Drosophila	NP_523546.1	
Alpha-2-delta Three mutually exclusive exons	Between VWA and Cache domains	Crassostrea	XP_011447859.2	QEY..DSL
		Crassostrea	XP_011447858.2	QAY..DPR
		Octopus	KOF93326.1	QHY..DSH
		Octopus	XP_014768966.1	QEF..DRS
Insert exon in alpha delta	Between Cache domain and C-terminus	Crassostrea	XP_011447859.2	SADNDTAN

### Expression of RFP-tagged Ca_V_2α1 in *Aplysia* sensory neurons requires 48 h and is not enhanced with co-expression of Ca_V_β and Ca_V_α2δ

**Figure 2:**
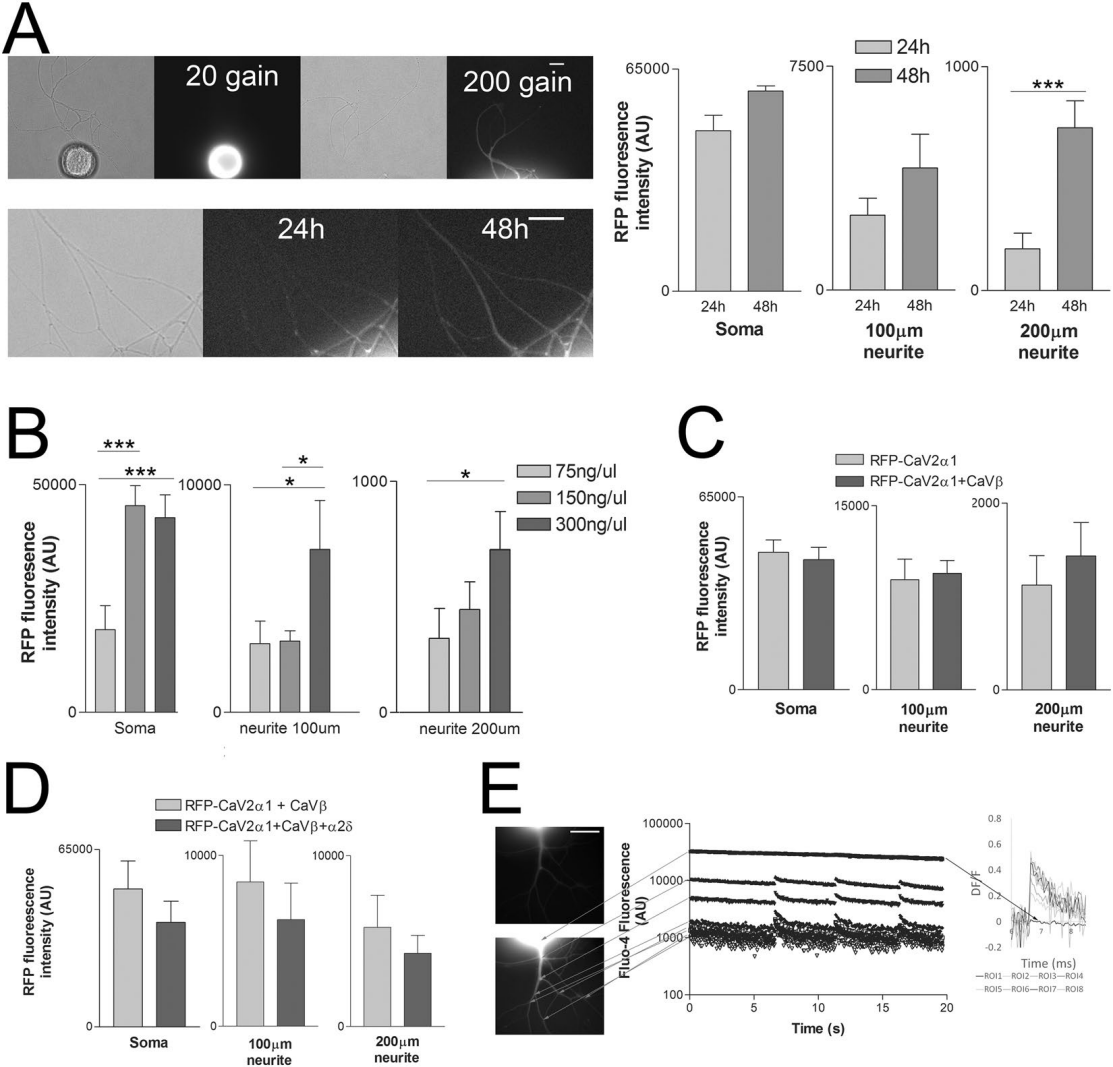
Expression of exogenous RFP-tagged Ca_V_2α1 subunits in sensory neurons. RFP-Ca_V_2α1 expressed for 48 h was imaged in the soma and in the neurites at approximately 100 μm and 200 μm from the soma. (**A**) Increasing the duration of RFP-Ca_V_2α1 expression from 24 h to 48 h significantly increases mean RFP fluorescence intensity in the distal neurites ~200 μm from the soma (n = 10, 10 sensory neurons, ***is P < 0.001 comparing 24 h to 48 h with paired t-tests, P = 0.0770 in the soma, P = 0.1415 at 100 μm, P = 0.0003 at 200 μm). (**B**) Increasing the plasmid injection concentration increased expression in the soma until 150 ng/μl, whereas further increasing the plasmid concentration to 300 ng/μl did further increase expression in the neurites (n = 13, 15, 21 sensory neurons, *is P < 0.05 and ***is P < 0.001 comparing groups with a one-way ANOVA and Bonferroni post tests). Specific F and P values are as follows for Soma F = 10.294, 75–150 P = 0.0005, 75–300 P = 0.001, 150–300 P = 1.0, for 100 μm neurite F = 5.148, 75–150 P = 1.0, 75–300 P = 0.031, 150–300 P = 0.029, for 200 μm neurite F = 5.038, 75–150 P = 1.0, 75–300 P = 0.017, 150–300 P = 0.072. (**C**) Co-expression of the RFP-Ca_V_2α1 subunit with the *Aplysia* Ca_V_β subunit has no effect on RFP intensity in the soma or out in the neurites (n = 15, 21, all comparisons made with t-tests, P = 0.6701 in the soma, P = 0.7915 at 100 μm, P = 0.5327 at 200 μm). (**D**) Co-expression of RFP-Ca_V_2α1 with *Aplysia* Ca_V_β and *Aplysia* Ca_V_α2δ, did not further increase expression in the soma or out in the neurites. Imaging conditions (excitation intensity and exposure time) were very different between soma and neurite imaging, thus values in the soma are not comparable with neurite values (n = 6, 8 sensory neurons, all comparisons made with t-tests, P = 0.3770 in the soma, P = 0.5306 at 100 μm, P = 0.4607 at 200 μm). All values are background subtracted 16-bit arbitrary units, and soma and neurite imaging conditions were very different and are therefore not comparable. (**E**) Representative Fluo-4 fluorescence imaging of three single action potentials. Top image is RFP signal from RFP-Ca_V_2α1, and the bottom image is Fluo-4 that is enhanced to see neurites. Fluo-4 intensity at different ROIs reveals measurable transients in the neurites, but no measurable transient in the soma. At the far right, the ROIs are converted to ∆F/F values to show transients of similar amplitudes in the neurites, but no measurable transient at the soma. Scale bars are 20 μm.

We generated an N-terminal RFP-tagged Ca_V_2α1 and expressed the subunit in cultured *Aplysia* pleural sensory neurons. RFP expression is clear in the soma and weak but present out in the processes (Fig. 2). Expression intensity in the neurites, particularly at neurites ~200 μm from the soma increased with 48 h expression compared to 24 h expression, thus 48 h expression was used in all other experiments unless otherwise stated (Fig. 2A). Changing the concentration of vector revealed that increasing from 75 to 150 ng/μl enhanced somatic fluorescence intensity, while intensity continued to increase in the neurites with a further doubling of the injected vector concentration to 300 ng/μl, thus this concentration was used in the rest of the experiments (Fig. 2B). Ca_V_2α1 transport to neurites could be limited by the availability of accessory subunits of Ca_v_2. However, co-expression of the wildtype RFP-Ca_V_2α1 with the *Aplysia* Ca_V_β subunit had no effect as measured with RFP intensity in the soma or out in the neurites (Fig. 2C). Similarly, coexpression of the RFP-Ca_V_2α1 subunit with the *Aplysia* Ca_V_β and the *Aplysia* Ca_V_α2δ subunits did not further increase RFP fluorescence intensity in the soma or in the neurites (Fig. 2D). Previous measurements of calcium influx in *Aplysia* sensory neurons demonstrated that endogenous Ca_V_2 channels are expressed in the neurites but not on the surface of the soma^11^. Despite the high intensity of the RFP-Ca_V_2α1 subunit in the soma, we could measure single action potential Fluo-4 transients only in the neurites as previously reported for endogenous currents (Fig. 2E). These experiments (and all other Fluo4 measurements) were done in the presence of Nifedipine (see methods) to remove any contributions of Ca_v_1 channels.

**Figure 3:**
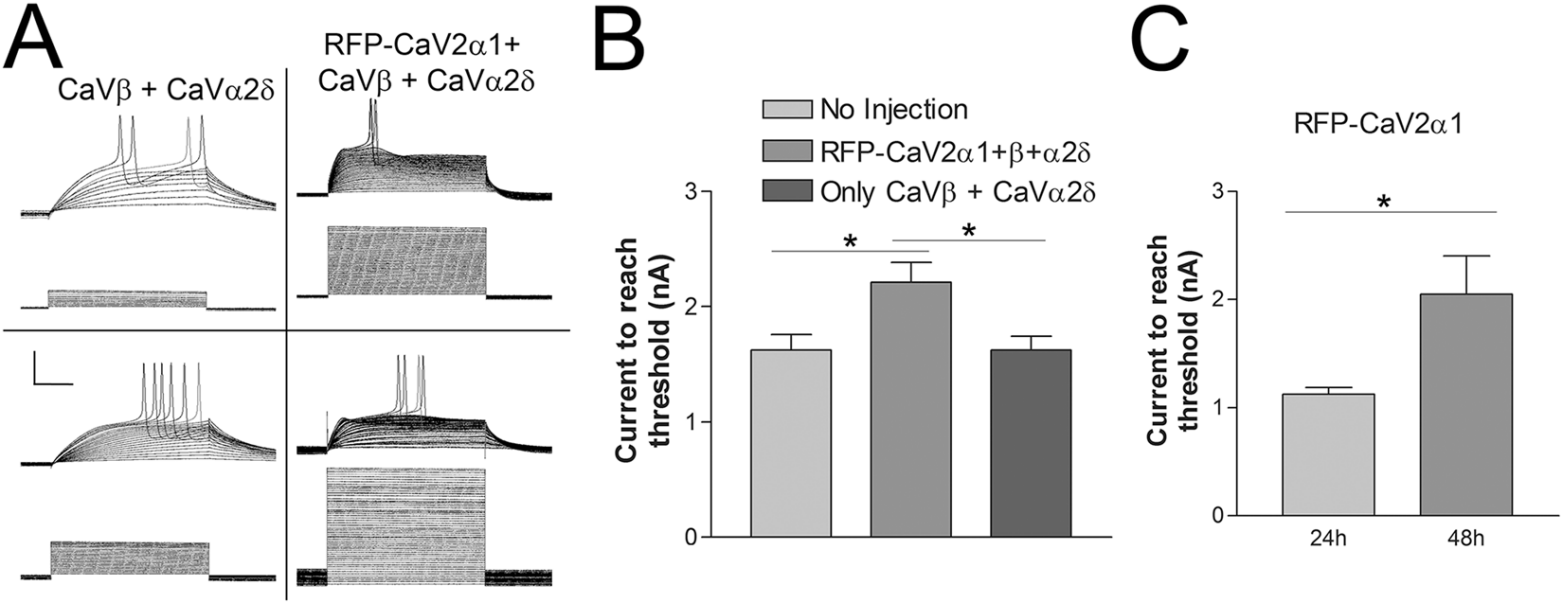
Reduction in membrane excitability with Ca_V_2α1 over-expression. (**A**) Traces of membrane potentials above the command currents from two sensory neurons expressing the Ca_V_β and Ca_V_α2δ subunits only (left column) and two sensory neurons expressing RFP-Ca_V_2α1 + Ca_V_β + Ca_V_α2δ. Depolarizing pulses are 100 ms at 1 Hz, scale bars are 40mV-1nA/25 ms. (**B**) The amount of current required to reach threshold was significantly increased with expression of the Ca_V_2α1 subunit, this was not seen with Ca_V_β and Ca_V_α2δ alone. N is 8, 10, 8 sensory neurons, *is P < 0.05 comparing means with a one-way ANOVA F = 5.393 with Bonferroni post tests, (P = 0.031 and 0.022 comparing RFP-Ca_V_2α1 + Ca_V_β + Ca_V_α2δ to No Injection and Ca_V_β + Ca_V_α2δ respectively). (**C**) The amount of current required to reach action potential threshold increased in sensory neurons with 48 h expression of RFP-Ca_V_2α1 (compared to 24 h expression with a t-test, P = 0.023, n = 11,12).

Electrophysiological examination of sensory neurons expressing RFP-Ca_V_2α1 subunits reveals a change in membrane excitability (Fig. 3A). SNs expressing exogenous Ca_V_2α1 require significantly more depolarizing current to reach action potential threshold (Fig. 3B). This change in membrane excitability was only observed with expression of a Ca_V_2 alpha 1 subunit; expression of accessory subunits alone did not change membrane excitability. A change in membrane excitability was not observed with 24 h expression when little RFP-Ca_V_2α1 was expressed in the neurites, while SN expressing RFP-Ca_V_2α1 for 48 h show a significant change in membrane excitability (Fig. 3C). This correlated with the increase in RFP intensity in the neurites. This change in excitability allows for monitoring of successful expression, but does not confirm that recombinant Ca_V_2α1 subunits are expressed on the cell surface and participating in voltage-dependent calcium entry, since the increase in excitability is unlikely to be a direct effect of calcium channel expression (see Discussion).

### Strong evolutionary conservation of the Ca_V_2α1 EF-hand F-helix tyrosine in Ca_V_2α1 subunits

**Figure 4:**
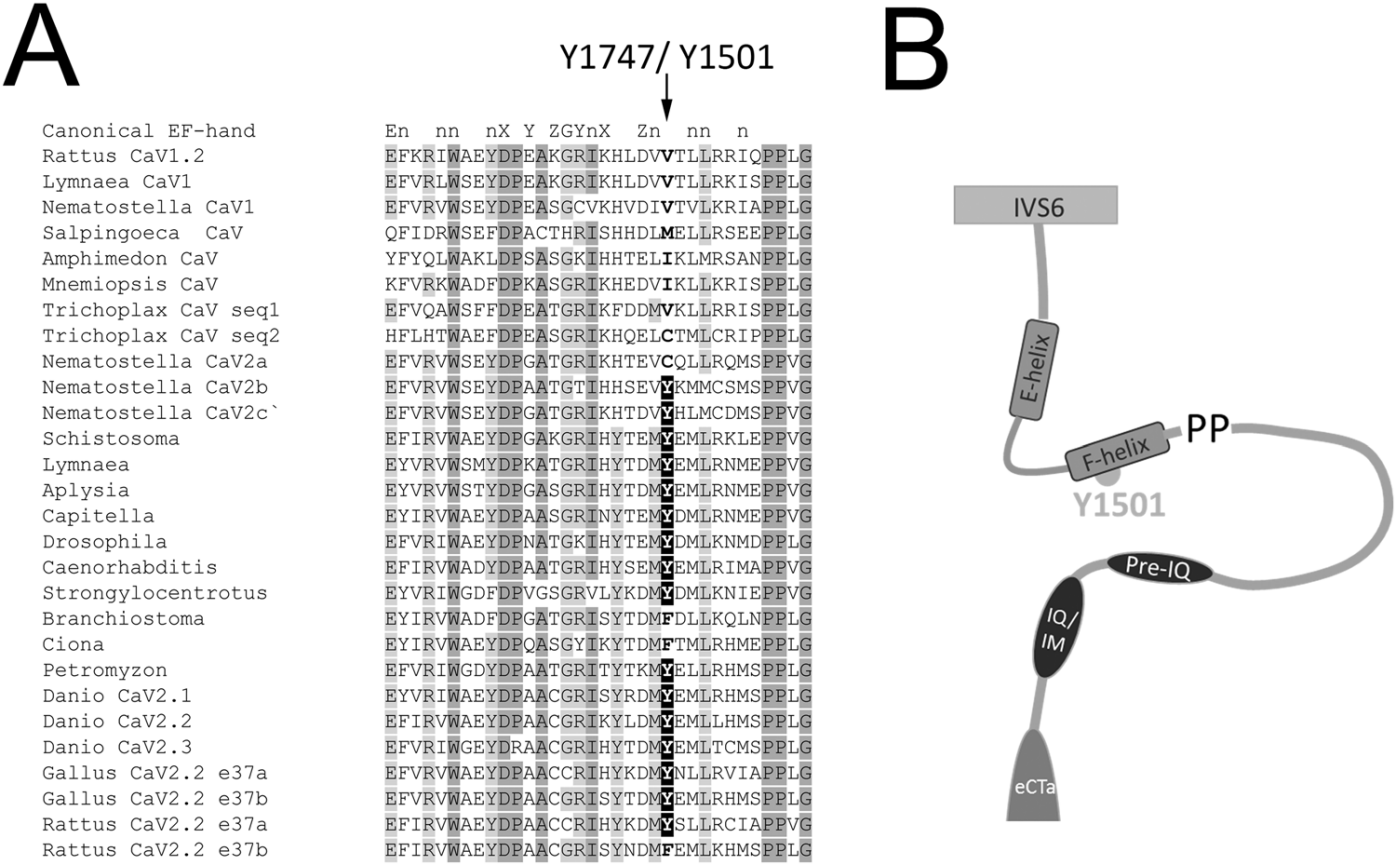
Ca_V_2 C-terminal EF-had alignment of sequences representative of the different Metazoan HVA calcium channels, highlighting the F-helix tyrosine. (**A**) Alignment of HVA Ca_V_ EF-hand sequences with three Ca_V_1 isoforms, the Choanoflagellate Ca_V_ channel, early metazoan HVA Ca_V_ channels, and representative Ca_V_2 isoforms from major Metazoan phyla. The conserved Ca_V_2 channel F-helix Y is highlighted in black. Note the Cnidarian Ca_V_2 duplications represented as *Nematostella* Ca_V_2a,b,c,; the loss of the conserved EF-hand F-helix Y in early Chordates; the vertebrate Ca_V_2 duplications as *Danio* Ca_V_2.1, 2.2, 2.3; and the loss of the conserved EF-hand F-helix Y to F with alternate exon e37b in Mammalia as *Rattus* Ca_V_2.2 e37b. The *Danio* (and *Xenopus*) Ca_V_2.2 gene has only a single exon e37, and while the Gallus Ca_V_2.2 gene does have two e37 alternate exons both e37a and e37b have the conserved tyrosine. (**B**) Cartoon showing the location of the EF-hand of the Ca_V_2α1 C-terminus and conserved F-helix Y relative to the final transmembrane sequence (IVS6), the highly conserved PP location of the *cac ts2* mutation^42,43^, IQ/IM domain^50^, and the alternate exon eCT.

We have previously shown that dopamine acting through an endogenous receptor and 5HT acting through expressed 5HT1A receptors inhibits the endogenous Ca_V_2 calcium current in *Aplysia* sensory neurons^10,11^. The inhibition through 5HT1A activation was blocked with the Src kinase inhibitor PP2, consistent with observations of voltage-independent inhibition of vertebrate channels occurring through Src kinase modulation^11^. This voltage-independent inhibition occurs in mammalian Ca_V_2.2 channels expressing exon e37a through tyrosine kinase phosphorylation of the EF-hand Y1747^15^. The tyrosine residue in the F-helix of the C-terminal EF-hand of Ca_V_2 is conserved in the *Aplysia* Ca_V_2α1 sequence, and comparison to other metazoan high-voltage activated calcium channels reveals that this residue is highly conserved throughout the evolution of the Ca_V_2α1 subunit (Fig. 4). The first animals with Ca_V_ channels, predating the differentiation of Ca_V_1 and Ca_V_2, do not have a tyrosine in the F-helix of the EF-hand (Fig. 4; i.e *Porifera*, *Ctenophora and Trichoplax*). The first evolutionary branch to have a recognizable distinction between Ca_V_1 and Ca_V_2 are Cnidarians, and Cnidarians have multiple Ca_V_2 channels, some of which contain the tyrosine (2 of 3 in *Nematostella* and *Acropora*, and 4 out of 10 for *Hydra v*.; Fig. 4). Thus, the tyrosine at this position first is observed soon after the divergence of the HVA- Ca_V_ channels into Ca_V_1 and Ca_V_2 early in Metazoan evolution, prior to the common Bilaterian ancestor. Indeed, almost all Bilaterian Ca_V_2 genes contain a Y, with an F occurring only in early Chordates (*Amphioxus* and the Urochordates, *Ciona* and *Oikopleura*) and mammalian Ca_V_2.2 channels expressing e37b. This data suggests that the Y of the F-helix of the Ca_V_2α1 C-terminal EF-hand is highly conserved throughout evolution and may underlie a conserved mechanism for modulation of Ca_V_2 currents and thus downstream evoked transmitter release. To test the hypothesis that the Src kinase regulation of *Aplysia* Ca_V_2 occurs through the *Aplysia* ortholog of the F-helix EF hand tyrosine, we generated a non-phosphorylatable mutant *Aplysia* RFP-Ca_V_2α1 Y1501F.

### *Aplysia* RFP-Ca_V_2α1 wt or *Aplysia* RFP-Ca_V_2α1 Y1501F with *Aplysia* Ca_V_β and *Aplysia* Ca_V_α2δ produce a Ca_V_2 current when expressed in HEK293t cells

**Figure 5:**
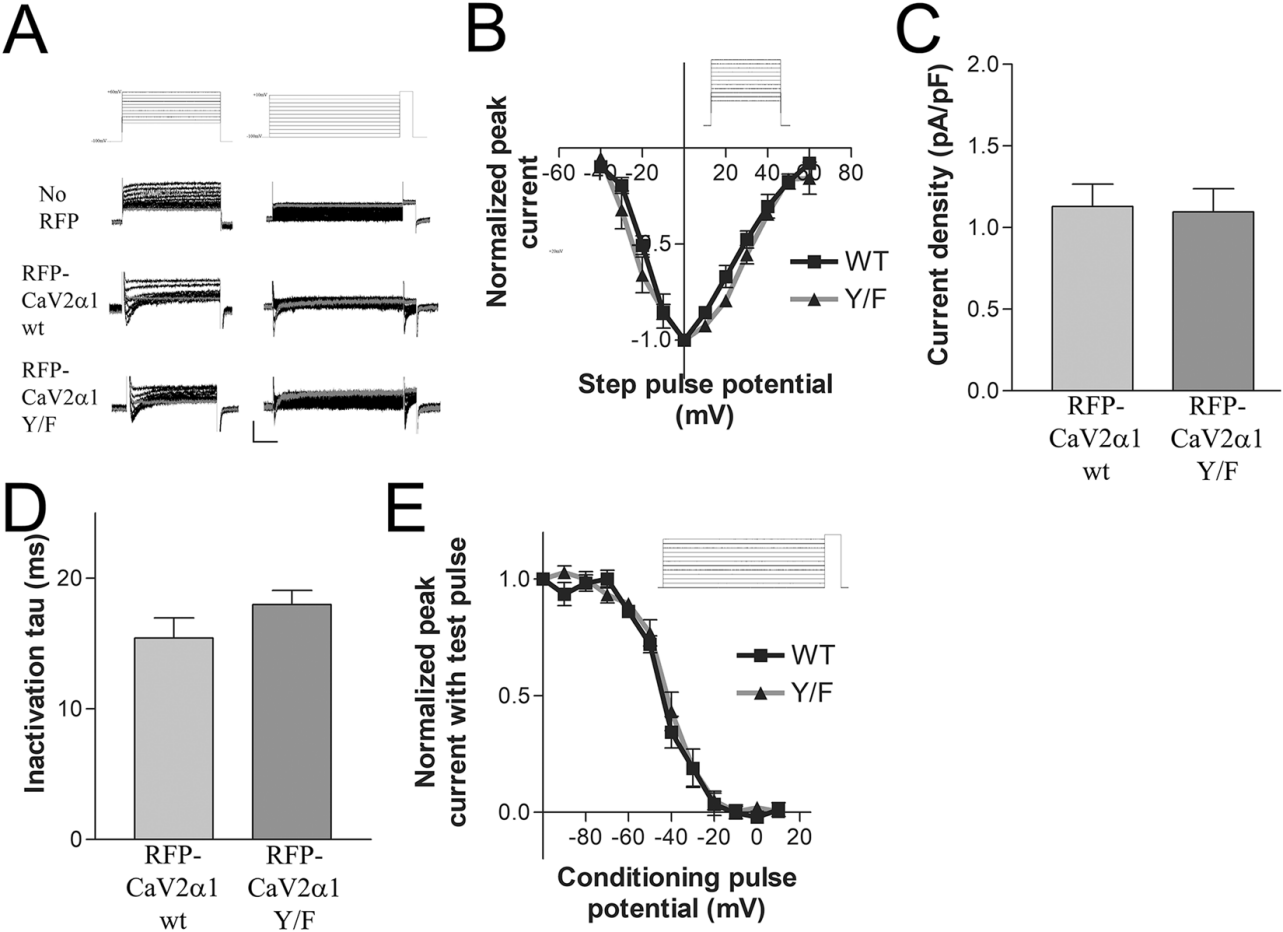
Whole-cell currents from HEK293t cells expressing *Aplysia* RFP-Ca_V_2α1 wildtype or RFP-Ca_V_2α1 Y1501F, and Ca_V_β and Ca_V_α2δ. (**A**) Representative traces from activation (left column) and steady-state inactivation (right column) voltage-clamp experiments. Top row are the command voltage steps overlaid, followed by the currents overlaid from representative cells without RFP, and RFP expressing cells transfected with either RFP-Ca_V_2α1 wt or RFP-Ca_V_2α1 Y1501F (along with Ca_V_β and Ca_V_α2δ). The grey trace is the voltage step to −40 mV in the activation protocol and to 0 mV in the steady-state inactivation protocol, scale bars are 20 pA, 50/200 ms. (**B**) Activation curves generated from the protocol displayed in A for RFP-Ca_V_2α1 (WT) and RFP-Ca_V_2α1 Y1501F (Y/F) normalized to the peak current for each group. Data included only for cells with a larger peak inward current than outward current at the end of the step, to remove trials with larger residual potassium currents (n = 4,4). One trial from the Y1501F group was removed from the averaged data as an outlier at >10 pA/pF. (**C**) Comparison of mean current density between cells expressing RFP-Ca_V_2α1 and RFP-Ca_V_2α1 Y1501F (t-test P = 0.8632, n = 10,10). (**D**) Comparison of mean inactivation tau of peak currents between cells expressing RFP-Ca_V_2α1 and RFP-Ca_V_2α1 Y1501F (t-test, P = 0.1990, n = 10, 10). (**E**) Steady-state inactivation curves generated with 1 s conditioning pulses followed by a step to the test potential of + 20 mV comparing cells expressing either RFP-Ca_V_2α1 (WT) or RFP-Ca_V_2α1 Y1501F (Y/F) (n = 7,7).

To ensure that the RFP-tagged wildtype Ca_V_2α1 subunit and the Y/F mutant both express on the cell surface and conduct a current consistent with a Ca_V_2 current we subcloned our channels subunits into vectors suitable for expression in HEK293t cells, a cell line derived from human embryonic kidney cells. Either *Aplysia* RFP-Ca_V_2α1 wt or *Aplysia* RFP-Ca_V_2α1 Y1501F expression along with the *Aplysia* accessory subunits *Aplysia* Ca_V_β and *Aplysia* Ca_V_α2δ generated inward barium currents consistent with other Ca_V_2 currents, including isolated endogenous *Aplysia* Ca_V_2 currents (Fig. 5 ^11^). The activation, inactivation and steady-state inactivation curves are similar for both wildtype and Y1501F mutant, thus both constructs code functional channels with similar kinetics that can express on the cell surface (Fig. 5). Incubation of HEK cells at 28 °C for at least 24 hr prior to recording is considered to increase current density^20^. Whole-cell recordings for RFP expressing cells did not result in useable currents with incubation at 37 °C (0.57 ± 0.15pA/pF for n = 6 RFP expressing cells with Ca_V_2 currents). While incubation at 28 °C doubled whole-cell current density, the measured currents were still very weak. This may relate to the natural operating temperature of the channels in the animal (~14 °C), which is much lower than that of other Ca_V_2 channels expressed in HEK cells. The activation, inactivation, and steady-state inactivation parameters of RFP-tagged wildtype and RFP-tagged Y1501F mutant channels expressed in HEK cells demonstrates that both of our constructs can produce functional Ca_V_2 channels and that the Y1501F mutation does not by itself cause a significant change in the properties of the Ca_V_2 channel.

### Expression of RFP-Ca_V_2α1 Y1501F in *Aplysia* sensory neurons inhibits the reduction in action potential calcium influx with 5HT1A activation and with dopamine

**Figure 6:**
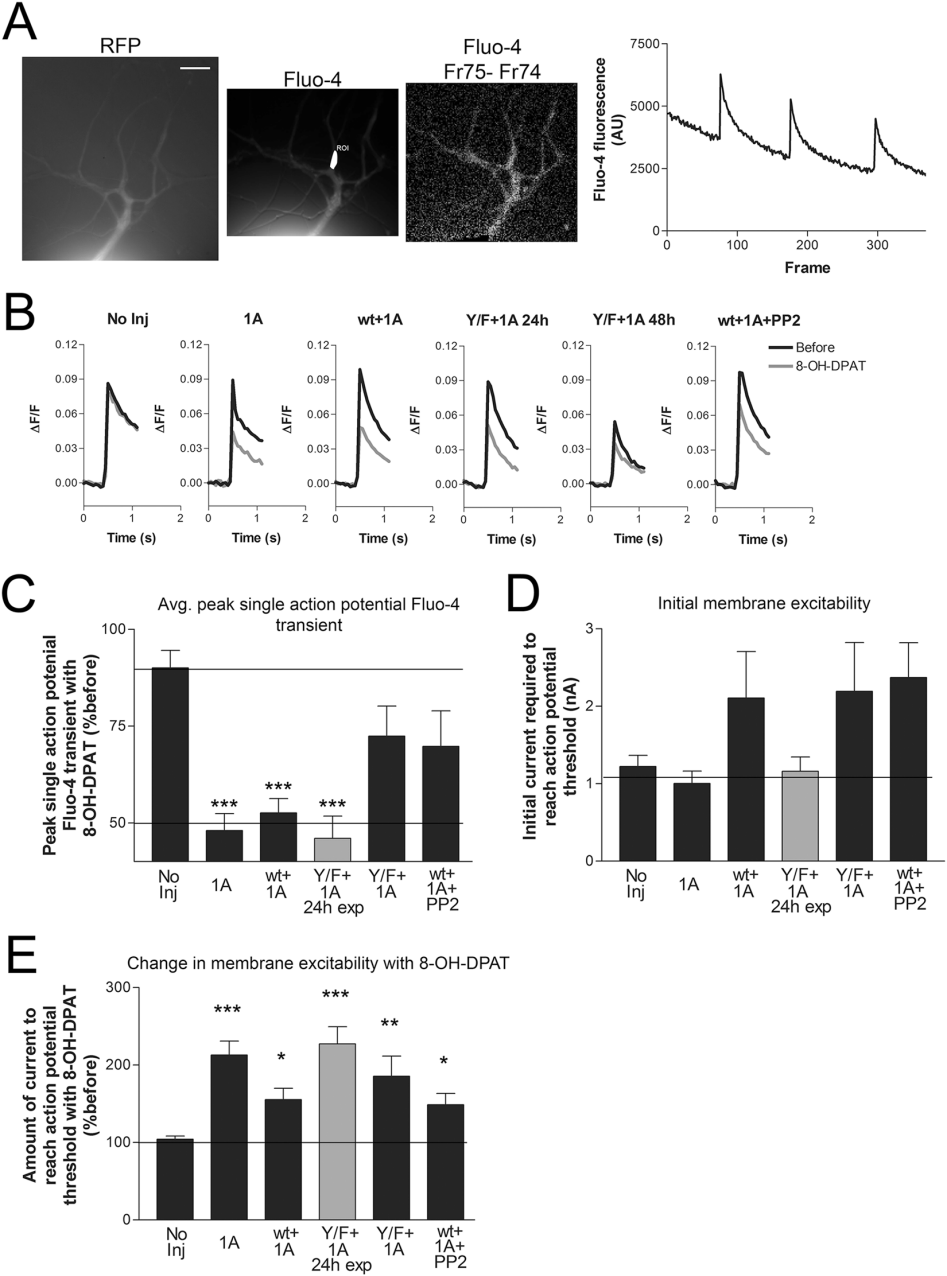
Inhibition of the sensory neuron single action potential Fluo-4 transient with 5HT1A expression & activation is inhibited with Src kinase inhibition or point mutation of the Ca_V_2α1 EF-hand F-helix tyrosine (Y1501F). (**A**) Images are RFP- Ca_V_2α1, Fluo-4 load with ROI selected, and Fluo-4 frame 74 before an action potential from frame 75 after an action potential approximately 50 ms later. On the far right is raw data for three single action potentials measured at the ROI marked in the middle image in white. The 20 μm scale bar on left panel applies to all the images. (**B**) Average of all single action potential Fluo-4 transients before and after application of 8-OH-DPAT for the five groups measured at 48 h and the one group 24 h post expression vector injection compared in CDE. The after measurement was made 30 s-1m after application of the 5HT1A agonist 8-OH-DPAT, whenever the change in membrane excitability was noted, and at one minute for the no injection group that do not respond to the agonist. Comparison of the initial peak Fluo-4 transient amplitudes (ΔF/F No Inj 0.089 ± 0.14, 1A 0.081 ± 0.018, wt + 1A 0.099 ± 0.023, Y/F + 1A 24 h 0.098 ± 0.031, Y/F + 1A 48 h 0.053 ± 0.008, wt + 1A + PP2 0.106 ± 0.009) between the different groups and the No Injection controls did not find any significant differences (a one-way ANOVA F = 0.731 and Dunnett’s post-test was used to compare groups to the No Injection control, P = 0.763, 0.921, 0.907, 0.373, 0.952, n = 7,6,7,5,7,7). (**C**) Summary of the change in the average single action potential peak Fluo-4 transient after 8-OH-DPAT application for the six different conditions. Five groups were measured after 48 h expression; No Injection, RFP + 5HT1A, RFP-Ca_V_2α1 wt + 5HT1A, RFP-Ca_V_2α1 Y1501F + 5HT1A, and a −20 min preincubation with PP2 and RFP-Ca_V_2α1 wt + 5HT1A. Another group was measured after only 24 h expression of RFP-Ca_V_2α1 Y1501F + 5HT1A. Groups expressing the 5HT1A receptor all had significantly (***P < 0.001) reduced mean Fluo-4 fluorescent transients with agonist except the group expressing RFP-Ca_V_2α1 Y1501F for 48 h and the group pretreated with PP2 compared to the ‘No injection’ group with an one-way ANOVA F = 7.206 and Dunnett’s post-test (n = 7, 6, 7, 5, 7, 7, comparing groups to No Inj P = 0.0002, 0.001, 0.0003, 0.181, 0.100). (**D**) Initial membrane excitability in the groups in B&C, measured as the amount of depolarizing current required to reach action potential threshold. As observed in Fig. 3, there was a trend for reduced excitability in the groups where an exogenous Ca_V_2α1 subunit was expressed for at least 48 h. However, the increases are not significant comparing groups to the No Injection control with a one-way ANOVA F = 1.896 and Dunnett’s post tests (P = 0.922, 0.249, 0.877, 0.243, 0.123). (**E**) All groups in B require significantly more current to reach action potential threshold with application of the 5HT1A agonist confirming 5HT1A expression & activation, compared to the ‘No injection’ condition with a one-way ANOVA F = 6.597 and Dunnett’s post test (Comparing groups to No Inj, P = 0.0003, 0.036, 0.00005, 0.005, 0.041). No sensory neurons in the No Injection group displayed the rare endogenous 5HT1A response.

The time required for cell adhesion to the dish, plasmid injection, and subsequent expression of the CaV2 constructs results in neurite outgrowth that is problematic for achieving an effective space clamp required for voltage-clamp analysis^11^. Thus, we used fluorescent calcium imaging to measure the calcium current. Despite the increased RFP expression seen at 48 h, expression of RFP-Ca_V_2α1 did not affect the peak amplitude of the Fluo-4 transients in the neurites (Fig. 6B). Although not significant, there was a trend for lower Fluo-4 transients when expressing the RFP-Ca_V_2α1 Y1501F channel (see Discussion), despite similar RFP expression with the two constructs (comparing wt to Y1501F in soma P = 0.3493, neurite 100 μm P = 0.2201, and neurite 200 μm, P = 0.4987, n = 10,6). The absence of a change in the Fluo-4 transient after expression of the channel could be due to the lack of surface expression of the exogenous calcium channel or constraints on calcium channel surface expression in this system. If there are constraints on calcium channel expression, then it is conceivable that the exogenous Ca_V_2α1 subunits might replace the endogenous Ca_V_2α1 subunits in the surface expressed Ca_V_2 channel. To explore this possibility, we compared the amount of heterosynaptic inhibition of the Fluo-4 transients without expressing a recombinant Ca_V_2α1, expressing RFP-Ca_V_2α1, and expressing RFP-Ca_V_2α1 Y1501F. Since in our previous study, only about 50% of pleural sensory neurons in culture respond to endogenous modulators^11^, we co-expressed the Aplysia 5HT1A receptor^9^. While, the *Aplysia* 5HT1A receptor is endogenously expressed in a very small percentage of pleural sensory neurons (~5–10%^10,21,22^), it can be exogenously expressed and subsequently activated with the agonist 8-OH-DPAT which strongly and consistently reduces both membrane excitability and the endogenous Ca_V_2 current, reducing variability in this experiment^11^. Inhibition of the *Aplysia* Ca_V_2 current with 5HT1A expression and activation was also found to be sensitive to the Src kinase inhibitor, PP2^11^. Thus, we compared the inhibition of the Fluo-4 transient with 5HT1A expression/activation in sensory neurons either with RFP-Ca_V_2α1 or the point mutant RFP-Ca_V_2α1 Y1501F. Application of the 5HT1A agonist 8-OH-DPAT without expression of 5HT1A (No Inj) show only a slight reduction in current. In contrast, when 5HT1A was expressed alone (1A) or with RFP-Ca_V_2α1 WT (wt + 1A), 8-OH-DPAT caused a statistically significant ~50% reduction in the peak single action potential Fluo-4 transient (Fig. 6BC). In contrast, when the Y/F point mutant RFP-Ca_V_2α1 Y1501F was expressed with 5HT1A (Y/F + 1A) or the Src inhibitor PP2 was present (wt + 1A + PP2), no significant inhibition was seen. This suggests that at least a portion of the 5HT1A inhibition of the Ca_V_2 current is through Src kinase, and requires a tyrosine in the F-helix of the EF-hand of Ca_V_2α1.

As described in Fig. 3, expression of exogenous Ca_V_2α1 subunits reduces initial membrane excitability as measured with the amount of current required to reach action potential threshold. The three groups expressing exogenous Ca_V_2α1 subunits for 48 h (RFP-Ca_V_2α1 + 5HT1A; RFP-Ca_V_2α1 Y1501F + 5HT1A; PP2 and RFP-Ca_V_2α1 + 5HT1A) required more depolarizing current to reach action potential threshold before addition of agonist (compared to the No injection, RFP + 5HT1A, and 24 h expression of RFP-Ca_V_2α1 Y1501F + 5HT1A groups) (Fig. 6D). Interestingly, normal heterosynaptic inhibition of the FLuo-4 transient with 8-OH-DPAT was seen with 24 h expression of RFP-Ca_V_2α1 Y1501F + 5HT1A, suggesting a correlation between the effectiveness of the mutant construct and the decrease in initial excitability. However, it is unlikely that the reduction in excitability could directly explain the decrease in heterosynaptic inhibition since the reduction in excitability was similar with 48 h expression of both RFP-Ca_V_2α1 WT and RFP-Ca_V_2α1 WT Y-F, but only the point mutant reduced heterosynaptic inhibition of the Fluo-4 transient. Another possibility is that the reduction in excitability is correlated with the surface expression of the calcium channel (See discussion).

Since expression of the channel had additional effects on sensory neurons, such as the change in excitability, one possibility for the lack of an effect of 5HT1A activation in the RFP-Ca_V_2α1 Y1501F + 5HT1A groups is a negative effect of the mutation on 5HT1A surface expression or signalling downstream of the receptor. An additional effect of signalling through 5HT1A is a decrease in membrane excitability. Indeed, the amount of current to reach action potential threshold was increased with application of the 5HT1A agonist 8-OH-DPAT, reducing membrane excitability in all groups injected with 5HT1A, confirming activation and signal transduction of the exogenously expressed 5HT1A receptors, and no effect of the Y1501F mutation on this expression (Fig. 6E). This also demonstrates that the mechanism by which 5HT1A reduces excitability is distinct from how overexpressing the calcium channel reduces excitability since the effects did not occlude each other. The effects of 8-OH-DPAT on membrane excitability were seen at 24 h, suggesting that, unlike the recombinant Ca_V_2α1, the 5HT1A receptor did not require 48 h of expression to be functional.

**Figure 7:**
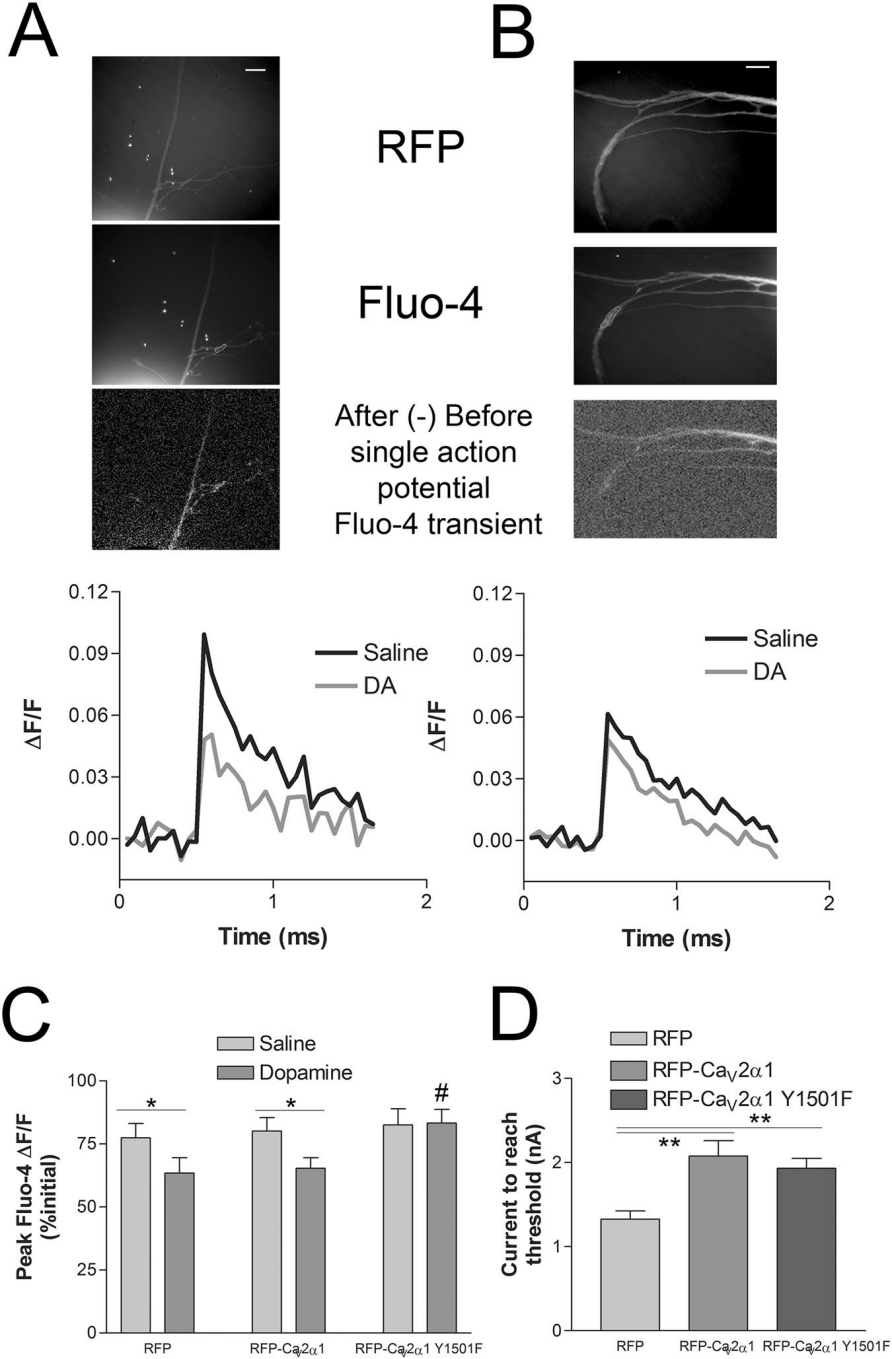
Single action potential Fluo-4 transients before and after dopamine. (**A**,**B**) RFP image used to choose Fluo-4 ROI prior to Fluo-4 load (upper images) and Fluo-4 image following loading with ROI indicated (middle images), image subtraction of the frame prior to action potential generation and the frame after (lower images). Representative ΔF/F Fluo-4 fluorescence transients from the trials depicted in images above. Averaged three consecutive single action potentials evoked at 0.2 Hz before and 1 min after application of 10 μm dopamine with SNs expressing either RFP-Ca_V_2α1 wt + Ca_V_β + Ca_V_α2δ (**A**) or RFP-Ca_V_2α1 Y1501F + Ca_V_β + Ca_V_α2δ (**B**). Scale bars are 20 μm. (**C**) Average peak Fluo-4 fluorescence transients to single action potentials as a percentage of the previous peak transients to account for run-down in the calcium transient. Addition of 10 μm dopamine significantly reduced the average single action potential peak Fluo-4 transients with RFP and RFP-Ca_V_2α1 wt + Ca_V_β + Ca_V_α2δ expression, but not with RFP-Ca_V_2α1 Y1501F + Ca_V_β + Ca_V_α2δ, comparing before to after dopamine with paired t-tests (*P < 0.05, n = 13,13,12). The RFP-Ca_V_2α1 Y1501F group was significantly different (P = 0.023) from the RFP alone controls using an one-way ANOVA F = 4.181 with Dunnett’s post test, ^#^P < 0.05 (P = 0.953, comparing wt to RFP alone controls). (**D**) Initial SN membrane excitability for the neurons used in C. Ca_V_2α1 over-expression as with either wt or Y1501F, but not RFP alone reduced membrane excitability, requiring more current to reach threshold with a 50 ms depolarizing pulse as in Fig. 3 (**P < 0.01, comparing wt (P = 0.001) and Y1501F (P = 0.003) to the RFP alone control with an one-way ANOVA F = 9.036 and Dunnett’s post test.

Dopamine can inhibit membrane excitability and the Ca_V_2 calcium current through activation of an endogenous receptor. To test whether the dopaminergic inhibition of Ca_V_2 also occurs through Y1501, we measured the single action potential Fluo-4 transients before and after application of dopamine in pleural sensory neurons expressing either RFP, RFP-Ca_V_2α1 wt + Ca_V_β + Ca_V_α2δ, or RFP-Ca_V_2α1 Y1501F + Ca_V_β + Ca_V_α2δ (Fig. 7A,B). Three single action potentials were evoked at 0.2 Hz before and 1 minute after addition of 10 μm dopamine. The average single action potential Fluo-4 fluorescence transients were significantly reduced in dopamine with RFP alone and with RFP-Ca_V_2α1 wt expression, but not with RFP-Ca_V_2α1 Y1501F (Fig. 7C). The weaker inhibition with dopamine compared to 5HT1A is likely due to the heterogeneity and variability in the endogenous dopamine response in pleural sensory neurons. The reduction in initial excitability with exogenous expression of Aplysia Ca_V_2α1 in SNs was again observed with both RFP-Ca_V_2α1 wt and RFP-Ca_V_2α1 Y1501F, confirming Ca_V_2α1 expression (Fig. 7D). Thus the inhibition of Ca_V_2 with dopamine also requires the conserved tyrosine in the F-helix of the EF-hand of Ca_V_2α1, indicating a common mechanism.

### The heterosynaptic depression with presynaptic 5HT1A activation is reduced with presynaptic expression of RFP-Ca_V_2α1 Y1501F

**Figure 8:**
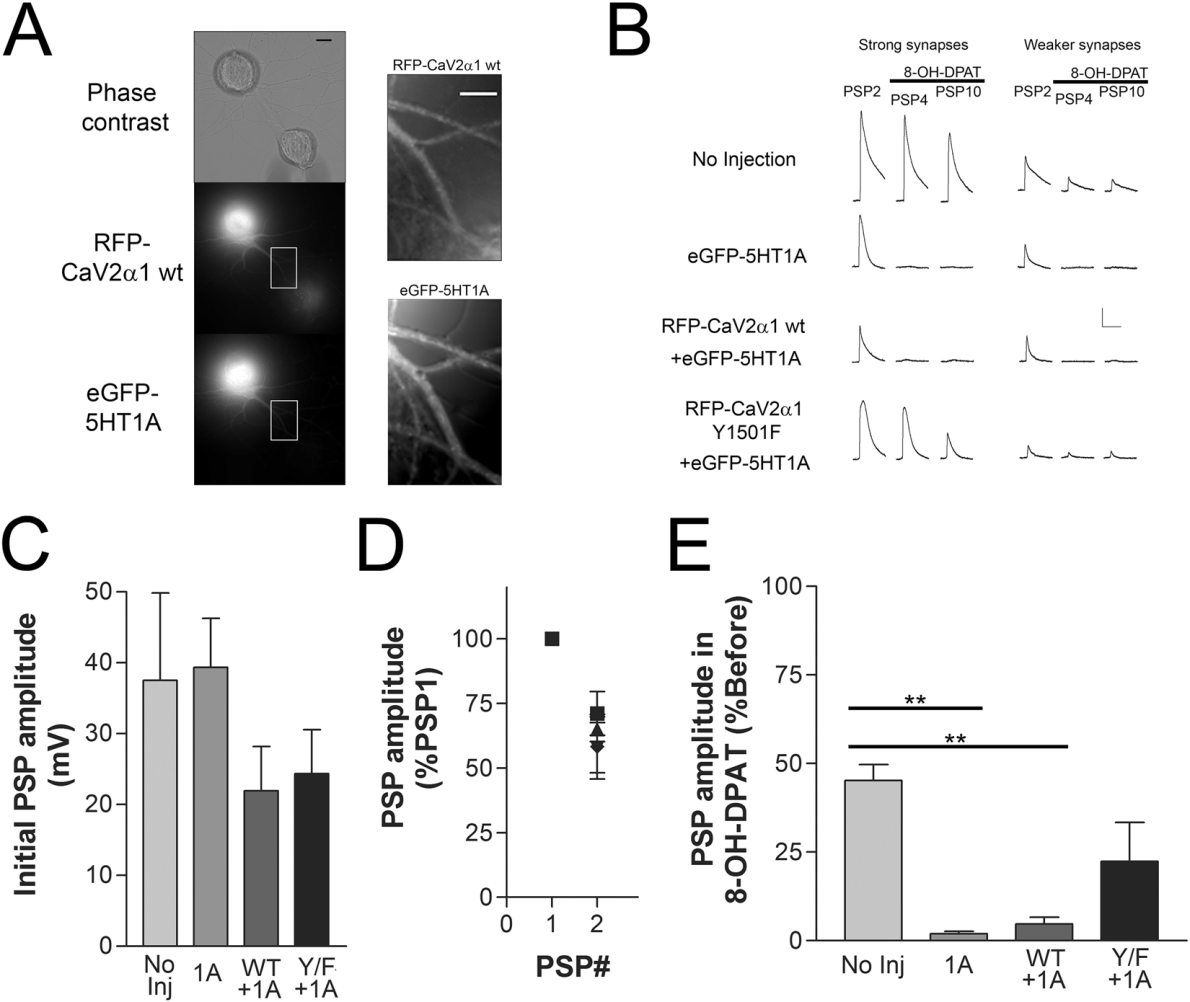
Expression of the Ca_V_2α1 Y1501F point mutant reduces heterosynaptic depression at *Aplysia* sensory to motor neuron synapses isolated in culture. (**A**) Images of RFP-Ca_V_2α1 wt and eGFP-5HT1A expression in a SN paired with a siphon motor neuron. Scale bars are 20 μm for the left column of images and 10 μm for the right column of images. (**B**) Representative traces of PSPs from the experiment summarized in CD (scale bar 10 mV/150 ms). PSP2 traces are prior to agonist, and PSP4 and PSP10 after agonist. Stronger synapses better describe the extent of the inhibition, however, larger PSPs activate voltage-activated currents that alter PSP amplitude and waveform. Initial synaptic strength varies, however both strong synapses (PSP > 20 mV) and weaker synapses (<20 mV) show the same pattern of inhibition. (**C**) Initial PSP amplitudes were similar between the four groups (comparing groups with a one-way ANOVA F = 1.265, P = 0.3077). (**D**) Two PSPs were evoked prior to addition of agonist, the second PSP was normalized to the first PSP revealing similar amounts of initial homosynaptic depression (comparing normalized PSP2 amplitudes between the groups with a one-way ANOVA where F = 0.3739, P = 0.7727). (**E**) Average PSP amplitude after application of 8-OH-DPAT (PSP4-PSP10, PSP3 data not included because of high variation in the onset of inhibition) as a percentage of the average PSP amplitude before agonist (PSP1 & PSP2). The 5HT1A agonist significantly reduces PSP amplitude at synapses expressing eGFP-5HT1A alone (1A, P = 0.005) and RFP-Ca_V_2α1 wt + eGFP-5HT1A (wt + 1A, P = 0.005), but not at synapses with the SN expressing RFP-Ca_V_2α1 Y1501F + eGFP-5HT1A (Y/F + 1A, P = 0.216) (data compared with a one-way ANOVA F = 6.596 with Bonferroni post-tests, n = 6, 6, 8, 9).

As the Ca_V_2 calcium current triggers action potential evoked neurotransmitter release and 5HT1A activation strongly reduces transmitter release we examined the role of Y1501F on heterosynaptic depression. Since we were not using Fluo-4 imaging we expressed an eGFP tagged 5HT1A receptor (eGFP-5HT1A) allowing visualization of the 5HT1A expression (Fig. 8A). Expressing eGFP-5HT1A with or without Ca_V_2α1 subunits did not significantly affect initial synaptic strength measured from the amplitude of PSP1 (Fig. 8C). Low frequency stimulation (<1 Hz) of *Aplysia* sensory to motor neuron synapses leads to homosynaptic depression of the postsynaptic potential (PSP) amplitude, a feature of the sensory to motor neuron synapse that is independent of heterosynaptic signalling^23^. The greatest amount of homosynaptic depression happens between PSP1 and PSP2, and this was not significantly affected with expression of either eGFP-5HT1A or the Ca_V_2α1 subunits (Fig. 8D, P > 0.05). Following the two PSPs to measure initial synaptic strength and depression we added 8-OH-DPAT and measured eight more action potential evoked PSPs at 0.2 Hz. At synapses in the No Injection group (no 5HT1A expression) average PSP amplitude continued to depress after application of 8-OH-DPAT such that average PSP amplitude was about 45% of PSP1, the result of continued homosynaptic depression (Fig. 8E). Expression of the 5HT1A receptor (1A, eGFP-5HT1A) in the presynaptic SN coupled to the application of 8-OH-DPAT leads to significantly increased depression in the PSP amplitude measured in the postsynaptic motor neuron compared to the level of homosynaptic depression seen in the absence of eGFP-5HT1A expression (**P < 0.01). However, co-expression of the point mutant RFP-Ca_V_2α1 Y1501F with eGFP-5HT1A reduced the inhibition in PSP amplitude with 5HT1A agonist application (Y/F + 1A, P > 0.05) (Fig. 8E). This suggests that the recombinant channels are being expressed at synapses, participating in transmitter release, and that the reduction in the Ca_V_2 current with 5HT1A activation contributes to heterosynaptic depression.

## Discussion

Early Metazoans such as the sponge *Amphimedon* and the comb jellyfishes *Mnemiopsis* and *Pleurobrachia* have a single gene coding the alpha 1 subunit of a high voltage-activated calcium channel as does the sister group to the Metazoans, the Choanoflagellates. In well sequenced Cnidarian genomes there is a clear Ca_V_1 gene and many Ca_V_2 genes, thus Ca_V_1 and Ca_V_2 diverged before the Cnidarian branch and early in Cnidarian evolution the Ca_V_2 gene was then duplicated multiple times^24^. All well sequenced Bilaterians have a single Ca_V_1 gene and a single Ca_V_2, with the exception of the vertebrates where genome duplication has led to four CaV1 and three CaV2 genes^25,26^. The tyrosine at 1747 first appears in Cnidarians, where many of the Cnidarian Ca_V_2α1 sequences have the conserved tyrosine. Y1747 (Y1501 in Aplysia) is conserved in most Bilaterian Ca_V_2α1 subunits with very few exceptions. Thus, the F-helix EF-hand tyrosine is highly conserved, a feature of most Ca_V_2α1 subunits.

The phosphorylation of Y1747 of vertebrate calcium channels with Src kinase leads to rapid VI-inhibition of the current^15,19^. The Src kinase-mediated inhibition of the N-type calcium current can be regulated in mammalian neurons through the mutually exclusive alternate exon pair e37a and e37b, where the F1747 containing e37b channels are insensitive to Src kinase inhibition^15,17^. The first indication that this may represent a conserved mechanism was our demonstration that the strong and consistent VI-inhibition of the *Aplysia* Ca_V_2 current with activation of the 5HT1A receptor is blocked with a Src kinase inhibitor^11^. Here we show that this inhibition requires the equivalent tyrosine as Src-kinase dependent inhibition of the mammalian channel. The similarity in the extent of the block of 5HT1A inhibition of the single action potential Fluo-4 transient with PP2 and Y1501F suggests a few points of note. Firstly, it suggests that our over-expressed point mutated alpha 1 subunits are getting out to the plasma membrane participating in voltage-mediated calcium entry and largely replacing endogenous Ca_V_2α1 at 48 h, a quality that is not measurable with the N-terminal RFP tag. Secondly, the inhibition of the *Aplysia* Ca_V_2 current in sensory neurons mediated by Src kinase activity requires Ca_V_2α1 Y1501. This is most likely through phosphorylation of this residue by Src, but at present we lack the reagents to directly measure phosphorylation of this residue and its dependence on Src. Expression of Ca_V_2α1 Y1501F not only prevented inhibition of calcium currents with dopamine and 5HT1A activation in isolated sensory neurons, but also reduced the decrease in transmitter release mediated by 5HT1A receptor activation at sensory-motor neuron synapses. The partial reversal of heterosynaptic depression with expression of Ca_V_2α1 Y1501 indicates that the heterosynaptic inhibition of transmitter release, at least partly, occurs through the inhibition of the Ca_V_2 current. Our experiments cannot rule out additional mechanisms independent of Ca_V_2α1 Y1501 that also contribute to heterosynaptic depression. Previous examination of heterosynaptic depression with FMRFamide implicated p38 MAP kinase and arachidonic acid in heterosynaptic depression^27,28^, while the mechanisms of this inhibition are not understood, similar pathways may combine with Ca_V_2 inhibition to mediate heterosynaptic depression with 5HT1A activation or alternatively, be involved in the activation of Src. The strong conservation of the Y1501 residue in most Ca_V_2 alpha 1 subunits with very few exceptions implies that this mechanism is ancient and we would predict that even Cnidarian channels could be regulated through this mechanism.

The 5HT1A receptor is expressed on the cell surface and can be activated with 24 h expression (Fig. 6C^22^), but RFP-Ca_V_2α1 Y1501F is not effective at preventing the inhibition of the calcium current at this same time point. The change in efficacy of RFP-Ca_V_2α1 Y1501F on Fluo-4 inhibition with the increase in the duration of expression from 24 h to 48 h also coincides with an RFP signal in the distal neurites and a change in membrane excitability. This indicates that the functional expression of Ca_V_2α1 requires additional time, potentially a result of the highly regulated trafficking and surface expression previously noted for these channels^29,30^. Consistent with the somatic exclusion of Ca_V_2 channels in *Aplysia* sensory neurons^11^, the action potential Fluo-4 transients of expressed recombinant RFP-Ca_V_2α1 subunits with the Ca_V_1 blocker nifedipine are only measured in the neurites despite the strong RFP signal in the soma. Whatever the mechanism that allows somatic expression of Ca_V_1α1 but excludes Ca_V_2α1 is not overwhelmed with Ca_V_2α1 over-expression. The coincident change in membrane excitability observed at all SN expressing an exogenous Ca_V_2α1 protein expressed for at least 48 h appears to relate to Ca_V_2α1 surface expression, however the mechanism for this is uncertain. Ca_V_2 channels should not open near SN resting potentials (and SNs are not spontaneously active neurons). Nor does it appear to be related to increased overall surface expression of Ca_V_2 channels with expression of recombinant channels since the initial Fluo-4 transients were not increased with Ca_V_2α1 expression (Fig. 6B legend). One possibility is that it involves co-trafficking of recombinant Cav2a1 subunits with a potassium channel and may relate to other examples of channel co-trafficking^31,32^. It is also possible that expression of the channel titrated out a repressor of surface expression of the channel controlling excitability, but it is unclear why this would occur at 48 hr, but not 24 hrs.

While we attempted to enhance RFP-Ca_V_2α1 surface expression with coexpression of the accessory subunits (β, α2δ) as suggested in other systems^29,33–35^, endogenous accessory subunits (β, α2δ) appear sufficient for the substitution of the endogenous/exogenous alpha 1 subunits as the efficacy of Y1501F mutant was similar with both 5HT1A inhibition (Fig. 6- only Ca_V_2α1) and dopamine inhibition (Fig. 7- Ca_V_2α1 along with Ca_V_β and Ca_V_α2δ). This is further supported by the change in membrane excitability and distal neurite RFP expression with 48 h expression of Ca_V_2α1 isoforms that was also unaffected by coexpression of the Ca_V_β and Ca_V_α2δ subunits. Thus, the factors that tightly regulate and limit Ca_V_2 surface expression involve more than just subunit availability in these neurons.

Ca_V_2α1 Y1501F expression consistently resulted in weaker initial single action potential Fluo-4 transients even though the Fluo-4 loads were similar between groups and experiments (Fig. 6B). While the difference was not significant with appropriate statistical analysis, reduced current amplitudes with Y1501F would be consistent with the reduction in current density reported for this residue with rodent channels^15,17,36,37^. We did not find a significant difference in the current density between Aplysia wildtype and Y1501F channels when expressed in HEK293t cells but this may relate to the overall poor expression of the channel in that system. Prolonged agonist exposure is known to lead to both GPCR and calcium channel internalization^38,39^, however when the kinetics of GPCR agonist-mediated internalization are measured, significant changes generally take at least a few minutes and tend to peak after thirty minutes^40,41^. Furthermore, the internalization of GPCR does not require the downstream G-proteins, unlike the rapid voltage-independent inhibition where heterotrimeric G-protein signalling is required^15,39^. If internalization of *Aplysia* Ca_V_2 underlies the inhibition with 5HT1A and dopamine, then the kinetics of the mechanism are on the scale of seconds rather than minutes. The reduction in current density with Ca_V_2.2e37b channels occurs through a reduction in surface expression, apparently through the disruption of an AP-1/AP-2 binding site with the loss of Y1747^37^. A similar mechanism may explain the reduced initial calcium transient amplitudes we observed but is less likely to contribute to the rapid phosphoregulation of the channel with Src kinase. In addition to reduced surface expression, the CaV2.2e37b channel (F not Y) also has differences from the e37a isoform that reduces current amplitudes through changes in channel gating kinetics^17,36^. Perhaps the change in CaV2.2e37b channel kinetics are mimicked with phosphorylation of Y1501, or the inhibition through phosphoregulation could occur through production of a new binding domain such as an SH2 or PTB, that leads to the loss or gain in a regulatory binding partner. The proximal Ca_V_2α1 C-terminal is known to be a site of channel kinetic regulation as the Drosophila Ca_V_2 paralytic mutation (*cac* ts2) occurs through a single amino acid point mutation 9 amino acids downstream of Y1501 by changing channel kinetics^42–44^. Furthermore, the Ca_V_1 calcium-dependent inhibition involves the EF-hand, as does the voltage-dependent inhibition through intracellular Mg^2+^, further highlighting the potential of this proximal C-terminal domains involvement in altering channel kinetics^45–47^. Further experiments will be needed to understand the mechanisms underlying the inhibition of the Ca_V_2 current with Src kinase. Given the many potential roles of Y1501, in surface expression, channel gating kinetics, and phosphoregulation, it remains unclear which of these features has led to the strong evolutionary conservation of this residue.

Overall, this initial work using the cloned *Aplysia* Ca_V_2 calcium channel demonstrates that we can functionally replace endogenous Ca_V_2α1 subunits with recombinant proteins and confirms that the Ca_V_2α1 subunit EF-hand tyrosine Y1501 (orthologous to rat N-type Y1747) is a target for regulation by GPCRs through Src. These experiments also demonstrate that the inhibition of the Ca_V_2 calcium current is at least partially responsible for the inhibition of neurotransmitter release with heterosynaptic depression.

## Methods

### Cloning of Ca_V_2 alpha 1 subunit, Ca_V_ beta subunit, Ca_V_ alpha 2-delta subunit

A tentative sequence of Ca_V_2α1 was assembled from transcriptome data (www.aplysiagenetools.org) based on finding transcripts with homology to the *Lymnaea* Ca_V_2α1 sequence^48^. No transcript in this database crossed two large GC rich regions (one poly G sequence in the large C-terminal splice and the other approximately 100–200 bp before the stop codon). We first cloned the 5′ end of Ca_V_2.2 from the start codon until the I-II llinker (1.2 kB) into pNEXmRFP to generate a fusion protein with monomeric RFP attached to the cytoplasmic N-terminal of Ca_V_2α1. The 5′ end was cloned with primers containing a KpN I site at the 5′ end and a BlpI site at the 3′ end to allow cloning into pNEX3mRFP (Table 2).

**Table 2:** Primers used in the cloning and generation of recombinant calcium channel subunits.

Name of primer	Sequence	Notes
CaV2a1 5′ end forward	GGGGGTACCATGGCCACATTTCAGGCCA	KpnI site
CaV2a1 5′ end reverse	GGGGCTCAGCCCTCTCCTCACTCAGGATCAC	BlpI site
CaV2a1 B forward	CCTTCTTCAAACTTCGCAGGCA	
CaV2a1 B reverse	GGGGCTCAGCGCACAATGAAGTAGGAAGAGGCCA	BlpI site
CaV2a1 C forward	CTTGACGGGCGAGGATTGGA	
CaV2a1 C reverse	GGGGCTCAGCTTGGGCATGAACCGACACA	BlpI site
CaV2a1 D forward	ACGTCGTCTTCTTCATCGTCT	
CaV2a1 D reverse	GGGGCTCAGCTCACTGGGAGGCATAAG	BlpI site
CaV2a1 Y-F	GGGGAATCCACTACACAGACATG*TTT*GAGATGTTGAGAAACATG	BamHI site Y-F in italics
CaV2a1 E forward (O)	CAAGATGGGGCCAGCGGAGA	
CaV2a1 E forward (I)	TGAGGGAGACAATCAGAAAACT	
CaV2a1 E reverse (O)	GGGAGTGACGGAGATCTGGC	
CaV2a1 E reverse (I)	GGGGCTCAGCGCCAAGCTCCGCCCTTGAGT	Blp I site
CaV2a1 F forward	CCGGCCCGAGCACGCCTC	
CaV2a1 F reverse	GGGGCTCAGCGTGGGCGTGGCGAGATG	Blp I site
CaV2a1 G forward	CGTCGTCGTCCCAGCCCATG	
CaV2a1 G reverse	GGGGCTCAGCCTGTCGTTAACACCAGTC	BlpI site
CaV 5′ end beta forward (O)	TGTAGCTCTCTCAGTCGCCTG	
CaV 5′ end beta forward (I)	GTGACATGCGCATCATCAAAACAG	
CaV 5′ end beta reverse (O)	GCGAGGGAGGCATCATGTTG	
CaV 5′ end beta reverse (I)	CAGTAGGCCTCCAGGAACTCG	
CaV 3′ end beta forward (O)	AACTGGCACAGTGTGCAC	
CaV 3′ end beta forward (I)	GATCTTGGATGAGAATCAGCT	
CaV 3′ end beta reverse (O)	GAGGGCTACATTATGCCACTCG	
CaV 3′ end beta reverse (I)	GGTGCTATATGTCTATACTGCCGT	
CaV 5′ end alpha2-delta forward (O)	CATACGGCCTTTGTGCAAG	
CaV 5′ end alpha2-delta forward (I)	TATGGCGGCGATCAGAACGA	
CaV 5′ end alpha2-delta reverse (O)	CGTCTCATTCTGGCTGAACTC	
CaV 5′ end alpha2-delta reverse (I)	CATCTTAAGGCAGGTATGG	
CaV 3′ end alpha2-delta forward (O)	CTACGCCTTCACGCCGA	
CaV 3′ end alpha2-delta forward (I)	CCTTCAGATTGGGCATCAG	
CaV 3′ end alpha2-delta reverse (O)	GTCGTTGCCATGGCCACA	
CaV 3′ end alpha2-delta reverse (I)	CCATCACCGACTGTCCGAT	

We then sequentially used PCR to clone fragments overlapping at their 5′ end with a unique restriction site in Ca_V_2.2 and with a 3′ BlpI site to insert them into the growing Ca_V_2.2 sequence (PCR primers are listed in table I). For the GC region in the large C-terminal splice (poly glycine stretch) we used nested PCR and then assembled the sequence in pJET2.1 before inserting back into the growing Ca_V_2.2 sequence. For the region containing the Src phosphorylation site, we first cloned the PCR fragment into pJET 2.1 (Thermo Fisher Scientific) and then used a primer containing the adjoining BamHI site to generate the tyrosine (Y) to phenylalanine (F) mutation (PCR primers are listed in Table 2). Similarly, PCR products with and without the large C-terminal splice were both used. Thus, four Ca_V_2α1 constructs were generated RFP-Ca_V_2α1, RFP-Ca_V_2α1 (Y1501F), RFP-Ca_V_2α1-ΔeCT, RFP-Ca_V_2α1-ΔeCT(Y1501F). For all the results shown, results with splice and no splice were combined as no significant differences were seen in expression, effects on excitability, or inhibition by modulatory inputs.

The Ca_V_ beta subunit (Ca_V_β) and alpha2-delta subunits (Ca_V_α2δ) were identified from transcriptome data (www.aplysiagenetools.org) using homology to the *Crassostrea* and *Lottia* sequences. For the Ca_V_β subunit, two PCR pieces (primers listed in Table 2) were cloned into pJET2.1 with a unique overlapping Mlu1 site and then assembled into one piece in pJET2.1 using MluI and NotI. The sequences was then excised from pJET2.1 with Xba and Bgl II and inserted into pNEX3 using Xba and BamHI. Similarly, the Ca_V_α2δ subunit, two PCR pieces (primers listed in Table 2) were cloned into pJET2.1 and then assembled in pJET2.1 using an overlapping SalI site and then the sequences excised with XhoI and XbaI and then inserted into pNEX3 cut with SalI and XbaI.

To express the channel in HEK293t cells, the channel was excised from pNEX RFP- Ca_V_2α1 with KpnI and NotI and inserted into pCDNA3-RFP at KpnI and Bsp120I (compatible with NotI). The joining site is identical since pNEX3-mRFP was generated from pCDNA3-mRFP. Ca_V_β and Cavα2δ were excised from pNEX3 with HindIII and KpnI and inserted into pCDNA3 at the same sites. Joinings were confirmed by sequencing.

Accession numbers for the sequences displayed in Fig. 4 are as follows; Rattus n. Ca_V_1.2 XP_008761438.1, Lymnaea s.Ca_V_1 AAO83839.1, Nematostella v. CaV1 XP_001639054.1, Salpingoeca r. XP_004989719.1, Amphimedon q. XP_011406227.1, Mnemiopsis l. AEF59084.1, Trichoplax a. s1 XP_002108930.1, Trichoplax a. s2 XP_002109775.1, Nematostella v. 2a ABAV01003013.1, Nematostella v. 2b ABAV01007705.1, Nematostella v. 2c ABAV01002357.1, Schistosoma m. AAK84311.1, Lymnaea s. AAO83841.1, Capitella t. 51958, Drosophila m. NP_001245638.1, Caenorhabditis e. NP_741734.1, Strongylocentrotus p. XP_011662956.1, Branchiostoma f. AAM18875.1, Ciona i. XP_018670105.1, Petromyzon m. ABQ96268.1, Danio r. 2.1 XP_017210285.1, Danio r. 2.2 XP_009299779.1, Danio r. 2.3 XP_017212913.1, Gallus g. 2.2e37a XP_015134748.1, Gallus g. 2.2e37b XP_015134761.1, Rattus n. 2.2e37a XP_008759800.1, Rattus n. 2.2e37b XP_006233631.1.

### HEK293t cell culture and electrophysiology

HEK293t cells were cultured in Dulbecco’s modified Eagle’s medium and 10% heat-inactivated fetal bovine serum (Invitrogen) containing penicillin and streptomycin^49^. Cells were transiently transfected with RFP-Ca_V_2α1 or RFP-Ca_V_2α1 (Y1501F), along with *Aplysia* Ca_V_β and Ca_V_α2δ at a plasmid ratio of 1:2:2 to increase the chances that RFP fluorescent cells also express the accessory subunits. Transfections were carried out using Polyfect (Qiagen) according to the manufacturer’s instructions. 24 h following transfection, cells were trypsinized and re-plated at a confluence of 30,000 to 60,000 cells per recording chamber dish and transferred to a 5% CO2 incubator at 28 °C for 48–96 h before recording. For electrophysiological recordings, the extracellular solution was composed of in mM: [BaCl2 20, MgCl2 1, CsCl 65, TEA 40, Glucose 10, HEPES 10, pH 7.2 with CsOH]. Whole-cell pipettes were fabricated to have resistances of 3–10 MΩ when filled with the internal solution composed of in mM: [CsMeSO3 108, MgCl2 2, ATP-Mg 2, EGTA 9, HEPES 9, pH 7.2 with CsOH]. Cells with RFP fluorescence were chosen for recording if membrane seal resistance was greater than 1 GΩ and access resistance less than 40 MΩ. Voltage-clamp control and current measurements were made with an Axopatch 200B and digitized with a Digidata 1322 A interface and pClamp software (Molecular Devices). Cells were held at −100 mV between voltage steps with 200 ms steps in the activation protocol and 1 s conditioning steps immediately followed by 100 ms test step to +20 mV for the steady-state inactivation protocol. Extracellular fluid was delivered to the cells through a SF-77B perfusion system (Warner Instruments) at a rate of 1 ml/min. All experiments were performed at 22 °C.

### Aplysia sensory neuron imaging and electrophysiology

*Aplysia californica* were obtained from the National Resource for Aplysia at the University of Miami. *Aplysia* pleural-pedal ganglia were digested in dispase II, desheathed, and ventral-caudal cluster pleural sensory neurons (SN) were individually isolated and transferred to poly-L lysine coated, glass bottomed culture dishes in a culture media containing 10% hemolymph and 90% isotonic L-15 media. With synaptic pairs, postsynaptic LFS siphon motor neurons were isolated from the abdominal ganglia based on position and morphology, and placed next to a single SN in a culture media containing 50% hemolymph and 50% isotonic L-15. After 24 h the sensory neurons were injected and left for 48 h before synapse measurements. Sharp glass microelectrodes ~15 MΩ in resistance when backfilled with 2 M potassium acetate were used for recording membrane potentials and to evoke action potentials. PSPs were evoked with single action potentials in the SN with 50 ms pulses with the motor neuron held at −80 mV. PSP amplitude was measured from peak amplitude at synapses <20 mV and initial PSP rise-rates for larger PSPs to avoid amplitude errors from non-synaptic voltage-dependent currents (apparent in some of the larger PSPs in Fig. 8, also see^11^). Electrodes were controlled with Sutter MP-225 micromanipulators and potentials amplified with an Axoclamp 900 A and digitized at 10 kHz with a Digidata 1440. The amount of current required to reach action potential threshold with a 50 ms pulse from a holding potential of −80 mV was used to measure membrane excitability.

Nuclear injections of pNEX expression vectors occurred after 24 h in culture and the neurons were then recorded/imaged 48 h after that, unless stated otherwise. Nuclear injections of DNA were through custom fabricated micropipettes using backing pressure regulated with a WPI PV820 picopump. DNA injections did not include fast green to avoid artifacts from spectral bleed through from the fast green into the RFP channel during fluorescence imaging. The recombinant 5HT1A and eGFP-5HT1A receptor sequences in pNEX3 vectors used were as described in^9,21^, and injected at a concentration of 200 ng/μL. PP2 (Sigma-Aldrich) was dissolved in DMSO at a final bath concentration of 0.1%, applied to the bath at a 10-fold concentration twenty minutes prior to the experiment. The 5HT1A agonist (±)-8-Hydroxy-2-dipropylaminotetralin hydrobromide (8-OH-DPAT) and dopamine (Sigma-Aldrich) were applied to the bath as a bolus with a pipette.

Fluorescence images were acquired on a Zeiss Axio Observer D1 through a EC Plan Neofluar 1.3NA 40 × oil coupled objective with a Photometrics QuantEM:512SC EM CCD camera. Standard Zeiss RFP filter sets (RFP 20) were used to image RFP intensity at maximum lamp intensity, constant exposure rate, CCD gain set at 20 for soma imaging and 200 for neurite imaging. RFP imaging was always done prior to Fluo-4 loading. For measuring RFP intensity in neurites, a circular ROI with a diameter equal to the width of the neurite was made at approximately 100 μm and 200 μm from the center of the soma. The mean background fluorescence measured at an adjacent ROI of the same size without any neurites was subtracted from the mean fluorescence intensity in the neurite. Fluo-4 pentapotassium salt (ThermoFisher Scientific) at 6 mM was backfilled in recording electrodes and iontophoretically injected into sensory neurons with hyperpolarizing pulses until sufficient intensity in the soma was reached (generally 5–10 min). Fluo-4 imaging was conducted with a standard Zeiss GFP filter (GFP 38) set and a lamp intensity of 25% and 25 ms exposure times with 2 × 2 pixel binning to achieve frame rates of ~20 Hz with minimal bleaching during imaging. Three single action potentials were evoked at 0.2 Hz with sharp microelectrodes before and 30 s to 1 min after agonist. Regions of interest were chosen at neurite branch points or varicosities with both an RFP signal and Fluo-4 fluorescence transient with an action potential. All calcium imaging experiments were done in the presence of 20 μm nifedipine (Sigma-Aldrich) added to the dish five minutes before the experiment. With the exception of Fig. 2E where raw Fluo-4 fluorescence values were used, all Fluo-4 transients were converted into ΔF/F_o_, where F_o_ was the average fluorescence values measured over the ten frames preceding the frame before evoking an action potential.

All images were analyzed using ImageJ and electrophysiological data analyzed with Clampfit. Statistical analyses were performed with Graphpad Prism and SPSS. All tests α = 0.05 and all data passed Kolmogorov-Smirnov normality tests prior to statistical comparisons. All data are means ± the standard error of the mean.

## Electronic supplementary material

Supplementary information
