# Supplementary material for: The CaV2α1 EF-hand F helix tyrosine, a highly conserved locus for GPCR inhibition of CaV2 channels
Source: Sci Rep. 2018 Feb 19;8:3263. doi: 10.1038/s41598-018-21586-5 (PMC5818475; doi:10.1038/s41598-018-21586-5)
Supplement: Supplementary file 1 — Supplementary information [file 41598_2018_21586_MOESM1_ESM.pdf]

Supplementary Information

The $Ca_V2\alpha1$ EF-hand F helix tyrosine, a highly conserved locus for GPCR inhibition of Ca_V2 channels

Tyler W. Dunn[1], Xiaotang Fan[1], Ariel R. Ase[2], Philippe Séguéla[2], Wayne S. Sossin[1*]

[1]Department of Neurology and Neurosurgery, Montreal Neurological Institute, McGill University, Montreal, Quebec, H3A 2B4, Canada.

[2]Department of Neurology and Neurosurgery, Montreal Neurological Institute, Alan Edwards Centre for Research on Pain, McGill University, Montreal, Quebec, H3A 2B4, Canada.

[*]Address correspondence to: Wayne S. Sossin, Department of Neurology and Neurosurgery, McGill University, Montreal Neurological Institute, Room BT 110, 3801 University Street, Montreal, Quebec, H3A 2B4, Canada. Tel.: 1-514-398-1486; Fax: 1-514-398-8106; E-Mail: wayne.sossin@mcgill.ca

For each alternative sequence, the cloned sequence is given first with the GENBANK accession number assigned to the cloned version of the calcium channel or associated subunit. Nucleotides and three letter code for amino acids are given for the region containing and surrounding the alternative sequence with the number of the starting nucleotide in the clone given. If the cloned sequence contains a region not found in other alternative sequences, these nucleotides and three letter codes are shaded green. Transcriptosome sequences are identified by the name given in Aplysiagenetools.org as these sequences have not yet been submitted to NCBI. In some cases, the sequence is entered in reverse orientation and this is noted, as well one of the alternative sequences was only found in an earlier database and this is also noted. These earlier databases are still publically available at Aplysiagenetools.org.

If the transcriptosome sequence contains a region not found in the clone sequence it is shaded in red. If we have confirmed the transcriptosome sequence by PCR it is noted in red text. The single letter codes for the cloned and alternative sequences are shown underneath. The order of alternative sequences corresponds to Fig. 1.

Splice site 1

GENBANK accession number KY705237

```
     Glu Glu Glu Glu Ala Val Arg Arg Glu Glu
2164 GAG GAG GAG GAG GCT GTG CGT CGG GAG GAG
```

Aplysia genetools.org TR68158|c8_g4_i8 len=5330
Confirmed by PCR

```
     Glu Glu Glu Glu Ala Val Asp Leu Leu Arg Arg Glu Glu
2484 GAG GAG GAG GAG GCT GTG GAC GAC CTG CGT CGG GAG GAG
```

EEEEAVRREE
EEEAVDLLRREE

Splice site 2

GENBANK accession number KY705237

```
     Ser Asp Thr Ala Ser Thr Thr Ser Ser
2462 TCT GAC ACT GCC AGC ACC ACC AGC AGC
```

Aplysia genetools.org CL929Contg1 len=6486

```
     Ser Asp Thr Ala Asn His Lys Pro Gly Ala Val Leu Gly
3902 TCT GAC ACT GCC AAT CAC AAA CCA GGG GCG GTC CTA GGA

Ala Ser Thr Thr Ser Ser
GCC AGC ACC ACC ACG AGC
```

Aplysia genetools.org comp83936_c0_seq11 len=4241
(pre 2014 assembly)

```
     Ser Asp Thr Gly Cys Trp Art Ala Art Ile Gly Phe Arg
1698 TCT GAC ACT GGC TGT TGG CGT GCC AGG ATT GGC TTC AGA

Ile Ala Ser Thr Thr Ser Ser
ATT GCC AGC ACC ACC AGC AGC
```

SDTASTTSS
SDTANHKPGAVLGASTTSS
SDTGCWRARIGFRIASTTSS

Splice site 3

GENBANK accession number KY705237

```
     Ala Phe Asn Glu Ser Ala Gly Lys Asn
2890 GCC TTT AAT GAG AGC GCG GGT AAG AAC
```

Aplysia genetools.org c128410_g2_i9 len=6174
Confirmed by PCR

```
      Ala Phe Asn Ala Phe Asp Ser Glu Ser Ala Gly Lys Asn
4051  GCC TTT AAT GCC TTT GAT AGT GAG AGC GCG GGT AAG AAC
```

AFNESESAGKNLN
AFNAFDSESAGKNLN

Splice site 4

GENBANK accession number KY705237

```
     Thr Ile Val Leu Met Met Lys Phe Asp Pro Lys Glu Ser Arg Gln Ser
3646 ACC ATC GTG CTC ATG ATG AAG TTT GAC CCC AAG GAA TCT CGC CAG TCC

Arg Arg Arg Glu Lys Gly Glu Asp Ala Ala Arg Iso Leu His Leu Ile Asn Thr
CGG CGC CGT GAG AAG GGG GAG GAC GCG GCC CGC ATC CTC CAT CTC ATT AAC ACG

Val Phe Thr Ser Leu Tyr Gly Leu Gly Phe Leu Leu Lys Leu Cys Ala Tyr Gly
GTC TTC ACC TCC CTC TAT GGC CTG GGG TTT CTG CTC AAG CTA TGC GCA TAC GGA

Lys Asn Tyr Phe His Asp Pro Trp Asn Val
AAG AAT TAC TTC CAC GAC CCC TGG AAT GTG
```

Aplysia genetools.org c128410_g2_i5 len=6135

Confirmed by PCR

Leu Asn Thr Iso Val Leu Met Met Lys Tyr Glu Thr Ser Asp Thr Tyr Lys Glu
AAC ACC ATC GTG CTC ATG ATG AAG AAA TAT GGC ACG TCG GAC ACA TAT AAG GAA

Val Leu Lys Tyr Leu Asn Ala Gly Phe Thr Ala Leu Phe Thr Iso Glu Cys Thr
GTC CTG AAA TAC CTA AAC GCA GGC TTT ACG GCT TTG TTC ACC ATA GAG TGT ACC

Ile Lys Iso Leu Gly Thr Gly Ala Arg Asn Tyr Phe His Asp Pro Trp Asn Val
ATC AAG ATA TTA GGC ACC GGT GCC AGG AAT TAC TTC CAC GAC CCC TGG AAT GTG

TIVLMMKFDPKESRQSRRREKGEDAARILHLINTVFTSLYGLGFLLKLCAYGKNYFHDPWNV
TIVLMMKYETSDTYKEVLKYLNAGFTALFTIECTIKILGTGARNYFHDPWNV

Splice site 5

GENBANK accession number KY705237

Ala Tyr Lys Ala Ser Gln Asn Ala Ser Asn Asn Phe Lys Met Arg Pro
4840 GCT TAC AAG GCC AGC CAG AAC GCT AGC AAC AAC TTC AAA ATG AGA CCG

Ser Leu Phe Arg Arg Leu Met Gly Gly Met Art Thr Ser Ser Ala Arg Ser Ser
TCT CTG TTC CGG CGA CTG ATG GGC GGC ATG CGG ACG TCC TCT GCC CGA TCC AGC

Gln Ser Leu Asp Ser Glu His Ser Asp Glu Asn Asn Gly Gly Gly Gly Gly Gly
CAG TCC CTG GAC TCT GAG CAC TCT GAC GAG AAC GAC GGA GGA GGG GGA GGA GGA

Gly Gly Gly Gly Gly Gly Gly Gly Gly Gly His Ser Phe Leu Arg Arg Asn Ser
GGA GGA GGA GGG GGA GGA GGA GGA GGA GGA CAT AGC TTC CTG CGC AGA AAT AGC

Ser Lys Arg Arg Arg Glu Ala Gly Asn His Gly Asp Ser Ser Asn Val Gln Pro
AGC AAG CGG CGG CGA GAG GCT GGC AAC CAC GGG GAC AGC TCC AAC GTG CAG CCC

Glu Arg
GGC AAC

Aplysia genetools.org _c3_seq72 len=3131

- reverse orientation
- Confirmed by PCR
- Used for expression

Ala Tyr Lys Ala Ser Gln Asn Ala Ser Asn Asn Phe Lys Met Pro
230 GCT TAC AAG GCC AGC CAG AAC GCT AGC AAC AAC TTC AAA ATG CCC

Glu Arg
GGC AAC

Aplysia genetools.org c128410_g2_i3 len=6177

- Confirmed by PCR

```
      Ala Tyr Lys Ala Ser Gln Asn Ala Ser Asn Asn Phe Lys Met Asn His
6001  GCT TAC AAG GCC AGC CAG AAC GCT AGC AAC AAC TTC AAA ATG AAC CAC

Asn Gly Lys Arg Pro Ser Leu Phe Arg
AAC GAA AAG AGA CCG TCT CTG TTC CGG
```

Aplysia genetools.org c128410_g2_i7 len=6183

- Confirmed by PCR

```
Trp Lys Ala Tyr Lys Ala Ser Gln Asn Ala Ser Asn Asn Phe Lys Met Asp Pro
TGG AAG GCT TAC AAG GCC AGC CAG AAC GCT AGC AAC AAC TTC AAA ATG GAC CCC

Val Arg Leu Pro Lys Arg Pro Ser Leu Phe Arg
GTC CGT CTG CCC AAG AGA CCG TCT CTG TTC CGG
```

Aplysia genetools.org GG9037|c0_g1_i1 len=313

- Confirmed by PCR

```
Trp Lys Ala Tyr Lys Ala Ser Gln Asn Ala Ser Asn Asn Phe Lys Met Asp Pro
TGG AAG GCT TAC AAG GCC AGC CAG AAC GCT AGC AAC AAC TTC AAA ATG GAC CCC

Val Arg Leu Pro Lys Asn His Asn Glu Lys Arg Pro Ser Leu Phe Arg
GTC CGT CTG CCC AAG AAC CAC AAC GAA AAG AGA CCG TCT CTG TTC CGG
```

WKAYKASQNASNNFKMRPSLFRRLMGGMRTSSARSSQSLDSEHSDENDGGGGGGGGGGGGGGGHSFLRRN
SSKRREAGNHGDSSNVQPGNDFSGG

WKAYKASQNASNNFKMPGNDFSGG

WKAYKASQNASNNFKMNHNEKRPSLFRRLMGGMRTSSARSSQSLDSEHSDENDGGGGGGG

- No transcriptosome sequence goes over the G rich region.

WKAYKASQNASNNFKMDPVRLPKRPSLFRRLMGGMRTSSARSSQSLDSEHSDENDGGGGGGG

- No transcriptosome sequence goes over the G rich region.

WKAYKASQNASNNFKMDPVRLPKNHNEKRPSLF

- No transcriptosome sequence goes over the G rich region.

No transcriptosome sequence found with inserts (NHNEK or DPVRLPK) without also having large splice.

Alternative Start 6

GENBANK accession number KY705239

```
       Met Arg Ile Lys Thr Glu Gly Val His Gly Ile Pro Gly Ser Met
 1     ATG CGC ATC AAA ACA GAA GGT GTC CAT GGG ATT CCC GGC AGC ATG
```

Glu Asn Ser Ser Pro Val Met His Gly Thr Leu Pro Gln Gly Arg Met Gly Arg
GAG AAC AGT AGC CCC GTG ATG CAC GAG ACG CTC CCC CAG GGC AGG ATG GGA CGC

Leu Arg Lys Gly Ala Thr Ser Gly Leu Ser Asp Ile Asp Ser Leu Gly Ser Ala
CTC AGG AAG GAG GCT ACC TCG GGC CTC AGC GAC ATC GAC TCC TTG GGT TCT GCA

Aplysia genetools.org TR114974|c3_g1_i1 len=1945

- reverse orientation
- Confirmed by PCR

Met Ala Leu Ala Pro Thr Gln Phe Gln Glu Phe Val Glu Thr Val Met
1547 ATG GCG CTC GCC CCA ACT CAG TTT CAG GAA TTT GTA GAG ACT GTT ATG

Pro Ser Ala Ser Gln Gly Lys Gly Gly Lys Arg Lys Gly Lys Phe Asn Lys Arg
CCA TCT GCT TCA CAG GGT AAG GGT GGC AAA CGC AAG GGC AAA TTC AAC AAA AGA

Ser Asp Gly Ser Phe Ser Asp Ser Ser Asn Ser Phe Ile Arg Gln Gly Ser Ala
TCG GAT GGG AGT TTC TCA GAC AGT TCT AAT AGT TTC ATT CGA CAG GGT TCT GCA

MRIKTEGVHGIPGSMENSSPVMHGTLPQGRMGRLRKGATSGLSDIDSLGSA
MALAPTQFQEFVETVMPSASQGKGGKRKGKFNKRSDGSFSDSSNSFIRQGSA

Alternative Splice 7

GENBANK accession number KY705238

Arg Val Gln Glu Tyr Ile Pro Val Leu Ser Arg Pro Gln
1282 AGA GTC CAG GAG TAT ATC CCT GTC CTG AGT CGG CCG CAG

Val Ile Lys Asn Lys Lys Thr Phe Glu Trp Gly Asn Ile Tyr Glu
GTC ATC AAA AAC AAG AAA ACC TTT GAG TGG GGC AAC ATT TAT GAG

Asp Thr Leu Gly Leu Gly Met Met
GAC ACT TTG GGG CTT GGC ATG ATG

Aplysia genetools.org comp122431_c4_seq37 len=4547

Arg Val Gln Asp Tyr Val Leu Val Leu Glu Arg Pro Leu Ala
1316AGA GTC CAG GAC TAT GTC CTC GTT CTA GAG CGG CCC CTG GCC

Leu Ala His Ile Glu His Glu Met Trp Thr Ser Ser His Lys Asn
TTG GCC CAC ATA GAA CAT GAA ATG TGG ACC AGT AGC CAC AAA AAT

Val Tyr Tyr Ala Lys Gly Gln Asn Pro Gly Leu Gly Met Met
GTG TAC TAC GCA AAA GGC CAG AAC CCG GGG CTT GGC ATG ATG

Aplysia genetools.org CL815Contig1 len= 5881

Arg Val Gln Asp Tyr Val Pro Val Leu Gly Arg Pro Gln

1315 AGA GTC CAG GAC TAT GTC CCT GTT CTG GGG CGG CCC CAG

Ala Ile Lys Asn Lys Lys Asn Lys Met Trp Thr Asn Ile Asn Glu
GCC ATC AAA AAC AAA AAA AAA AAA ATG TGG ACC AAC ATC AAT GAG

Asp Ile Ser Gly Leu Gly Met Met
GAT ATT TCG GGG CTT GGC ATG ATG

RVQEYIPVLSRPQVKLNKKTGEWGNIYEDTLGLGMM
RVQDYVLVLERPLALAHIEHEMWTSSHKNVYYAKGQNPGLGMM
RVQDYVPVLGRPQAIKNKKNKMWTNINEDISGLGMM

Alternative Splice 8

GENBANK accession number KY705238

     Arg Asn Asn Met Arg Ser Gln
2079 AGG AAC AAC ATG CGG AGT CAG

Aplysia genetools.org CL815Contig1 len= 5881

    Arg Asn Asn Ile Asn Asn Ser Thr Asn Ala Ser Asp Thr Gly
2113AGG AAC AAC ATA AAT AAT AGC ACA AAC GCG TCG GAC ACT GGC

Val Phe Ser Pro Leu Asp Asp Asp Asp Glu Arg Ser Gln
GTG TTC TCT CCC CTT GAT GAT GAT GAT GAG CGG AGT CAG

RNNMRSQ
RNNINNSTNASDTGVFSPLDDDDERSQ
